# A practical guide to self-organising maps in psychological research

**DOI:** 10.3758/s13428-026-03198-4

**Published:** 2026-11-12

**Authors:** Ido Shalev, Estherina Trachtenberg, Maria B. Jelen, William Mills, Marc Bennett, Rebecca P. Lawson, Tim Dalgleish, Duncan E. Astle

**Affiliations:** 1 MRC Cognition and Brain Sciences Unit, University of Cambridge, 15 Chaucer Road, Cambridge, CB2 7EF UK; 2 Department of Psychology, University of Cambridge, Cambridge, UK; 3 Department of Psychiatry, University of Cambridge, Cambridge, UK

**Keywords:** Self-organising maps, Machine learning, Unsupervised learning, Psychological methods, Person-centred approaches

## Abstract

Identifying shared and distinct profiles, whether of symptoms, personality traits, or cognitive abilities, is essential for understanding psychological phenomena, yet doing so requires navigating interrelated theoretical, conceptual, and technical complexities. First, most psychological phenomena typically exist along continuous spectra rather than discrete categories, complicating the characterisation of inherently heterogeneous constructs. Second, studying individual differences requires balancing population-level generalisation with person-level interpretability, which is critical for applied science and clinical practice. Third, psychological data often contain missing values, non-linear relationships, and high-dimensional patterns that violate standard statistical assumptions. Commonly used approaches for profile detection often fall short of these demands, producing oversimplified or difficult-to-interpret results that are hard to map back to the individual. In contrast, self-organising maps (SOMs) offer a powerful, visual, and intuitive solution that addresses these challenges. Despite growing interest, SOMs remain underutilised in psychology, likely due to a lack of accessible tutorials and clear guidelines tailored to the field. This tutorial introduces SOMs to psychologists without technical backgrounds, emphasising practical implementation. We provide evidence-based recommendations for key methodological decisions, accompanied by annotated R code. Using a dataset of depressive symptoms as an illustrative case, we present a complete workflow covering data preprocessing, map initialisation, parameter optimisation, and interpretation. We further discuss advanced SOM extensions relevant to psychological research and highlight key limitations of the method. By making SOMs accessible, this tutorial supports nuanced, person-centred analyses that preserve the complexity of psychological phenomena.

**Supplementary Information:**

The online version contains supplementary material available at https://doi.org/10.3758/s13428-026-03198-4.

Researchers across psychological sciences, whether studying development (Nordahl et al., 2022; Vandewouw et al., 2023), mental health (Brucar et al., 2023; Feczko et al., 2019; Gao et al., 2023), or social, emotional, and cognitive processes (Azari et al., 2020; Santavirta et al., 2024), often seek to identify profiles of individuals who share similar characteristics. Ideally, such profiles should preserve the structure of psychological constructs (De Boeck et al., 2023; Haslam et al., 2020) and remain interpretable at the individual level (Woo et al., 2024). However, commonly used methods (e.g., factor analysis, standard clustering) often fall short of these goals by imposing categorical assumptions that are not grounded in real-world observation (Haslam et al., 2020; Lahey et al., 2022) or producing abstract components that cannot easily be mapped back to individuals (Hope et al., 2024; Mazziotta & Pareto, 2019; Watts et al., 2023).

To illustrate, in mental health research, current classification systems often fail to capture heterogeneity within psychiatric disorders and similarities across them (Dalgleish et al., 2020), prompting efforts to reclassify disorders using data-driven methods (Conway et al., 2021; Forbes et al., 2025; Gao et al., 2023). For example, exploratory factor analysis (EFA) has been used to group co-occurring symptoms into broader factors (e.g., physical, cognitive, and affective; Puhalla et al., 2024; Ringwald et al., 2025; van Loo et al., 2022). Alternatively, principal component analysis (PCA) has been applied to reduce symptoms into fewer components capturing as much variability in symptoms as possible (Forbes et al., 2025; Franklyn et al., 2022; Stout et al., 2022).

In practice, however, psychologists are often interested in how variables combine *within individuals* (Bennett et al., 2025). Whether hopelessness in depression co-occurs with meaninglessness and preserved energy, or with fatigue and anhedonia, may change our assessment of suicide risk (Klonsky et al., 2018; Tondo et al., 2020). This person-centred perspective fundamentally differs from variable-centred methods like EFA, which assume a common factor structure *across* individuals, and from variance-centred methods like PCA, which transform variables at the cost of interpretability.^1^ Additionally, psychological phenomena rarely manifest as purely separated clusters (Haslam et al., 2020). Therefore, forcing dimensional data into rigid categories can obscure gradual transitions between constructs and distort how psychological processes are represented (De Boeck et al., 2023). In turn, this may reinforce a false dichotomy between categories (e.g., “healthy” versus “disordered”), which may contribute to stigmatisation by implying that groups of people (e.g., those experiencing mental health difficulties) are fundamentally different, rather than part of the same continuum (Johnstone, 2018; Lahey et al., 2022). What is needed, then, is a participant-centred approach that reveals how variables combine *within* individuals, and how those combinations are structured across the data, without reducing people to simply being members of a single group.

## Introducing self-organising maps

**Fig. 1:**
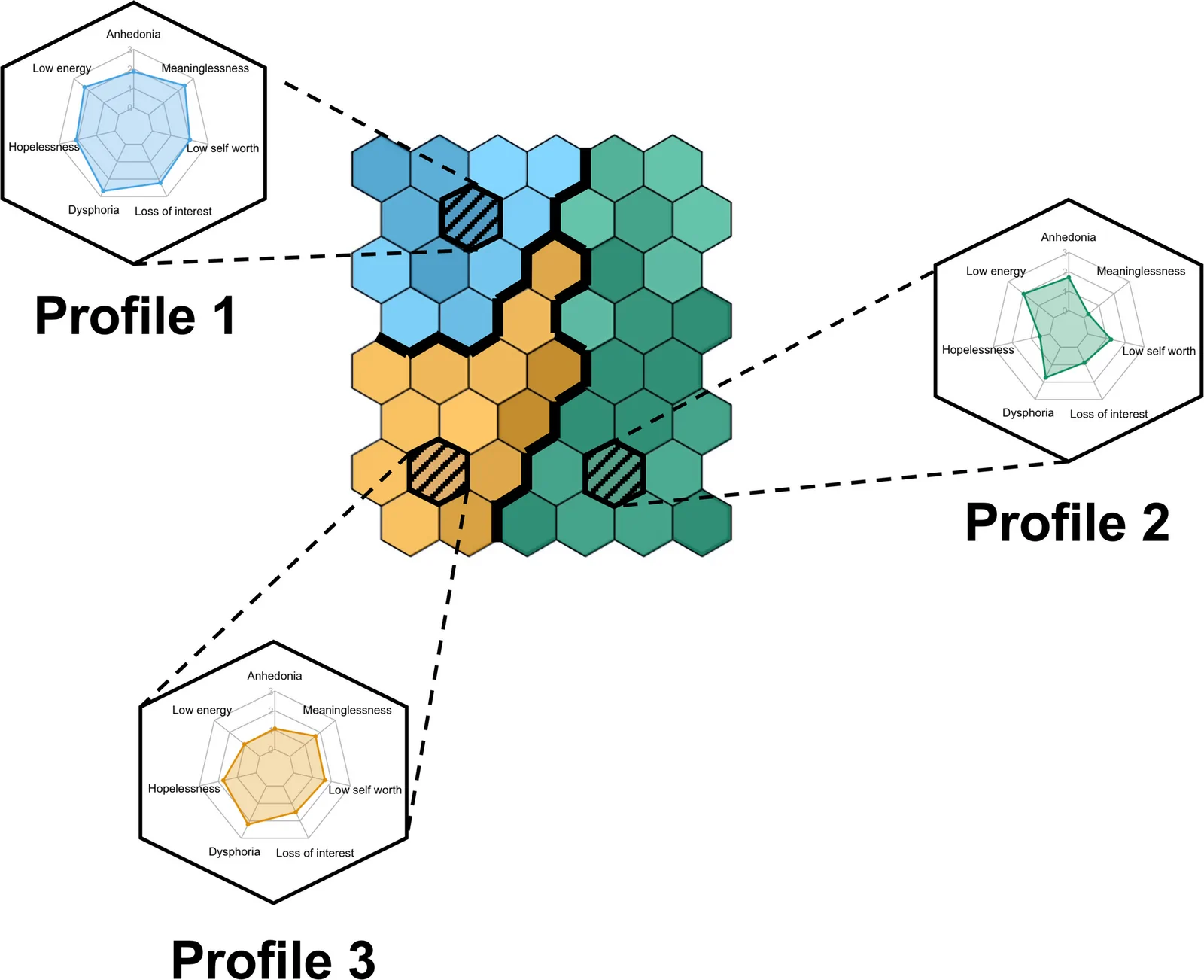
Example of a self-organising map. *Note*. Self-organising map based on seven depressive symptom items from the Depression Anxiety Stress Scales–21 (DASS–21). Each hexagon represents a group of individuals with similar symptom patterns. Groups with more similar patterns appear closer together on the map. The map reveals three main regions (blue, green, and orange), each representing a different depression profile. The radar plots display the most representative profile within each region (indicated by striped hexagons). Groups located closer to these representatives have profiles more similar to them, creating gradual transitions between regions. The black lines mark these transition points rather than hard categorical divisions. Accordingly, as the encircled radar plots illustrate, individuals in blue (Profile 1) tend to show high symptom levels across all domains. Individuals in green (Profile 2) tend to show relatively high fatigue, anhedonia, low self-worth, and dysphoria. Individuals in orange (Profile 3) are characterised by elevated hopelessness, meaninglessness, and dysphoria but relatively preserved energy levels. Scores are plotted on a 0–3 scale, where 0 (“*never*”) is at the centre of the radar plots and 3 (“*almost always*”) is at the outer edges, maintaining clear interpretation of the DASS–21, and reflecting how often each symptom was experienced over the past week

One powerful such method is self-organising maps (SOMs). SOMs are an unsupervised machine learning method that creates a two-dimensional map where *individuals* with similar patterns of variables are placed close together, while those who differ are farther apart (Kohonen, 2001). SOMs retain the original variables (and thus their interpretability), allowing researchers to examine each individual profile directly. At the same time, the map reveals how these profiles are organised in relation to one another, capturing the broader landscape of the data (Fig. 1). SOMs also capture non-linear patterns among variables (Yin, 2008), require fewer assumptions about data structure (Foody, 1999), and handle missing data more effectively than traditional approaches (Peeters & Dassargues, 2006). Together, these advantages make SOMs particularly valuable in psychology, where non-linear relationships and incomplete datasets are common (Failenschmid et al., 2025; Little, 2024), and where understanding heterogeneity within complex constructs is a central goal. To illustrate how SOMs differ from other methods, in the Supplementary Text, we apply four widely used methods (EFA, network analysis, PCA combined with *k*-means clustering, and latent profile analysis) to the same dataset used in this tutorial, enabling direct comparison. Table 1 summarises how these methods compare with SOMs.

**Table 1:** Summary comparison of methods

Method	Primary question	Approach	Strengths	Key limitations	How SOMs differ
EFA	How do items group into latent factors?	Variable-centred Dimensional Linear	Clear description of latent structure Foundational for scale validation	Assumes a uniform factor structure across individuals Cannot represent how people differ or how variables interact	SOMs are person-centred and retain each individual's full profile rather than reducing it to factor scores
Network Analysis	How do variables relate to one another?	Variable-centred Conditional associations (typically partial correlations) Linear	Can reveal fine-grained variable relationships Can identify "hub" variables	Cross-sectional networks remain variable-centred Silent on individual differences	SOMs map *people* rather than variables and thus complement networks rather than overlapping with them
PCA + *k*-means	Can participants be grouped on compressed item scores?	Person-centred Categorical Linear	Useful when many variables need to be reduced to a small number of components	Components are abstract and may be hard to interpret and communicate Hard categorical boundaries imposed by *k*-means	SOMs retain the original variables, capture non-linear structure, and replace hard boundaries with graded transitions
LPA	What probabilistic subgroups best fit the data?	Person-centred Categorical Multivariate	Quantifies classification uncertainty Uses items directly, thus preserving original variables’ meanings	Discrete categorical view May over-extract profiles when data is dimensional Assumes Gaussian within-profile distributions	SOMs are non-parametric and represent the latent structure as a continuous topology rather than a discrete set of profiles

SOMs are already being used to explore constructs including personality (Weingärtner Junior et al., 2024), emotional and cognitive processes (Khosrowabadi et al., 2010), and mental health conditions (Fekihal & Yousif, 2012; Galkin et al., 2022; Kristensen et al., 2024; Leiva-Murillo et al., 2013; Tervonen et al., 2020; Zou, 2022). For example, SOMs were used in a large cohort of over 8,000 people to identify four distinct profiles of individuals who had attempted suicide, distinguished by different factors (mental health disorders, alcohol problems, impulsiveness, or childhood maltreatment), suggesting that suicide attempts may arise through different routes for different people (Leiva-Murillo et al., 2013). In another study, a map was created to organise individuals without depression by their psychological profiles (based on variables such as attitudes towards life, sense of control, and social connections; Galkin et al., 2022). Individuals with depression were then placed on this map, and were over-represented in a specific area. Because the map changes gradually, the researchers could trace a continuum of changing attitudes from this area to the area most resilient to depression.

In autism research, SOMs have helped reveal distinct anxiety profiles in children, which are linked to differences in depression and peer-related difficulties (Leachman et al., 2024). In adolescent mental health research, SOMs have identified reproducible profiles ranging from flourishing well-being to widespread difficulties, with intermediate profiles showing focal internalising or externalising patterns (Bennett et al., 2026). They have also been used in children to uncover profiles of school-related challenges that were not predicted by children's diagnoses, suggesting that children with the same diagnosis can differ in their cognitive profiles (Astle et al., 2019; Siugzdaite et al., 2020). SOMs have further been used to classify cognitive task performance following working memory training (Rennie et al., 2020).

Despite these promising applications, SOMs remain underused in psychology, likely because existing literature assumes strong mathematical knowledge. Although introductory pieces (Oliver et al., 2018; Thuneberg & Hotulainen, 2006) provide helpful overviews, they lack step-by-step practical guidance tailored to psychological science.

This paper bridges this gap by providing a theoretical introduction and hands-on tutorial, written in accessible language, specifically designed for psychological research. To support this, we include a step-by-step script using the *kohonen* package (v3.0.12; Wehrens & Kruisselbrink, 2023) in R (v4.4.1; R Core Team, 2021), with an illustrative example providing all the essential information for researchers to implement SOMs in their work. For the illustrative case, we focused on depressive symptoms since individuals can exhibit 1,030 different symptom combinations (Fried & Nesse, 2015), necessitating a better understanding of the profiles within this diverse population. We selected 301 participants from a previously published study (Trachtenberg et al., 2024) who scored above threshold on the seven-item depression scale of the Depression Anxiety Stress Scales–21 (Lovibond, 1995).^2^ The example covers all stages, including data pre-processing, SOM training, interpretation and further analysis, visualisation, and recommended approaches for assessing validity and reliability.

## Understanding self-organising maps

**Fig. 2:**
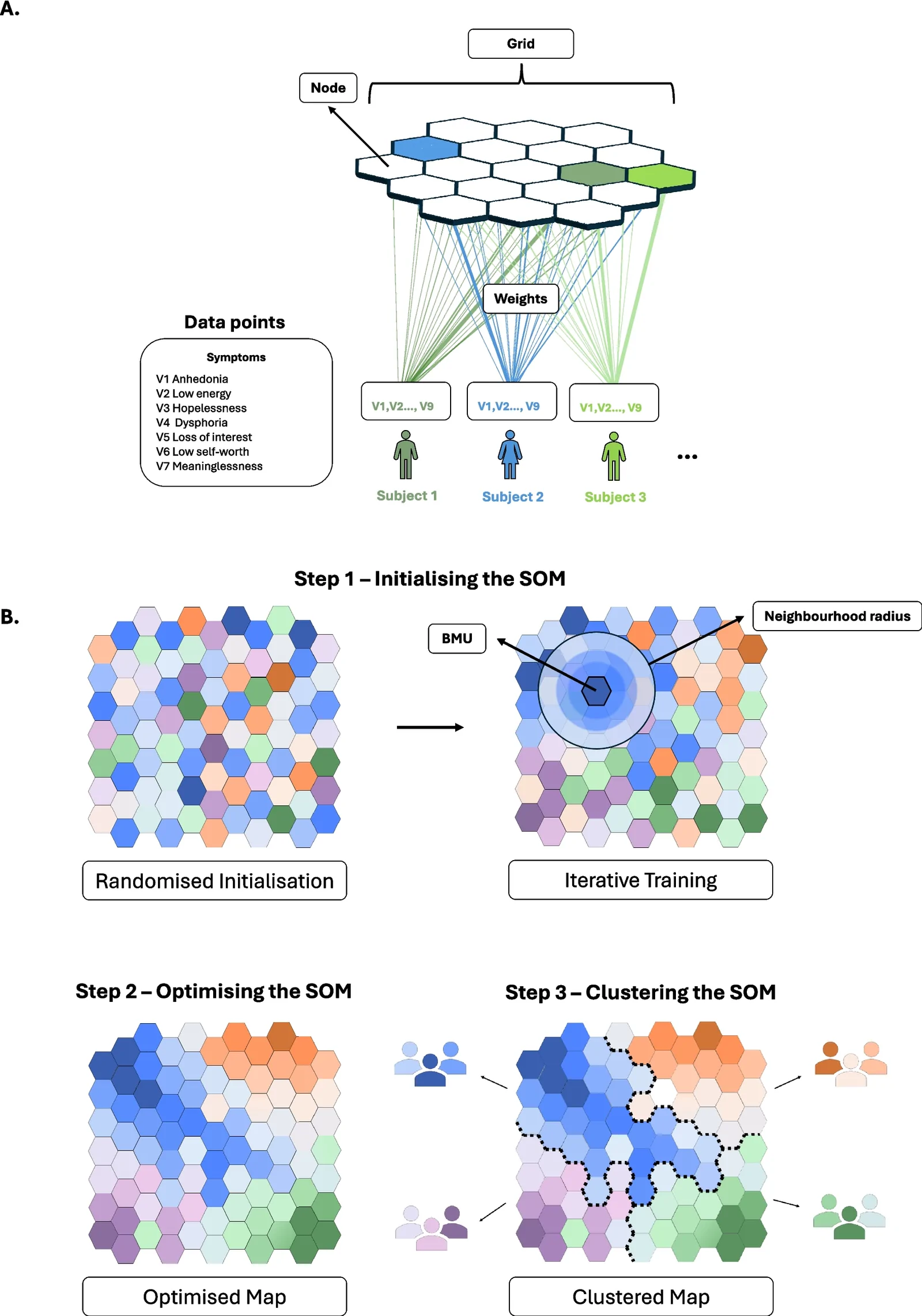
Structure and process of SOMs. *Note*. **A** Illustration of the structure of self-organising map (SOM), showing how data points, subjects with their individual symptom profiles (V1–V7), are mapped to a hexagonal grid structure. Each data point connects to nodes in the grid through weighted connections, with the grid serving as a topological representation, where individuals with similar patterns of depressive symptoms cluster together spatially. **B** Demonstrates the three main steps of the SOM analysis (initialisation, optimisation, and interpretation). During initialisation (step 1), the 301 participants with their seven depression scores would be fed into a randomly initialised grid. Through iterative training, each participant’s symptom profile would find its best matching unit (BMU) on the grid, with nearby nodes also being updated gradually within a defined neighbourhood radius. After training the initial SOM, participants who share similar depression symptom patterns (represented by similar colour of the node) will be clustered together, and fine-tuning optimisation of the SOM will be followed to minimise errors and improve the topology of the grid (step 2). The optimised map shows four distinct coloured regions (blue, purple, orange, and green) representing different depression symptom profiles that can be clustered together, with gradual transitions indicating related but distinct patterns (step 3). For example, one region might represent individuals primarily experiencing anhedonia and loss of energy, while another might capture those experiencing predominantly meaninglessness, hopelessness, and dysphoria. Within each region, some individuals (darker shade) may align with a more prototypical representation of the cluster, while others (lighter shade), particularly those near the boundaries, may show more mixed profiles

Figure 2 describes the structure and process of SOMs. A glossary summarising key terms (bolded at first mention) is included to support readers. At its core, SOM is a method for simplifying complex data while preserving the structure of relationships between similar observations. In other words, it reduces data with many variables into a two-dimensional map, called a **grid** (or feature map)**,** where individuals with similar patterns are positioned close together. Each point on the grid is called a **node**, representing a group of similar observations (called **data points**). In psychology, data points are usually participants, each with a set of variables of interest (e.g., depressive symptoms; Fig. 2A). Each node is assigned a random set of **weights**, with a single number for each variable in the data (Fig. 2A and B, *Randomised Initialisation*). Each weight is expressed on the same scale as its variable, and together a node's weights form its profile.

Through an iterative process, the SOM adjusts the weights to match the data structure. At each step, a data point is presented (e.g., seven symptom scores of one participant) and compared with the weights of every node. The node with the most similar weights, called the **best matching unit (BMU),** is identified. The BMU and its neighbouring nodes then update their weights to become more like that data point, with each weight moving a small step towards the participant’s score on that variable. Nodes closer to the BMU typically undergo larger updates than those further away (Fig. 2B, *Iterative Training*). This process repeats over multiple cycles (called **iterations** or **epochs**). Because each participant pulls the surrounding region of the map towards their own profile, neighbouring nodes gradually come to hold similar profiles. Early in training, updates are larger, establishing the broad layout of the map. Later, they become smaller and more local, fine-tuning individual nodes (see SOM parameters below). With each cycle, the map gradually organises itself so that similar data points end up near each other, creating a visual representation of the data’s underlying structure.^3^ By the end of training, each node's weights approximate the typical profile of the participants assigned to it, which is what researchers interpret.

## Practicalities

**Fig. 3:**
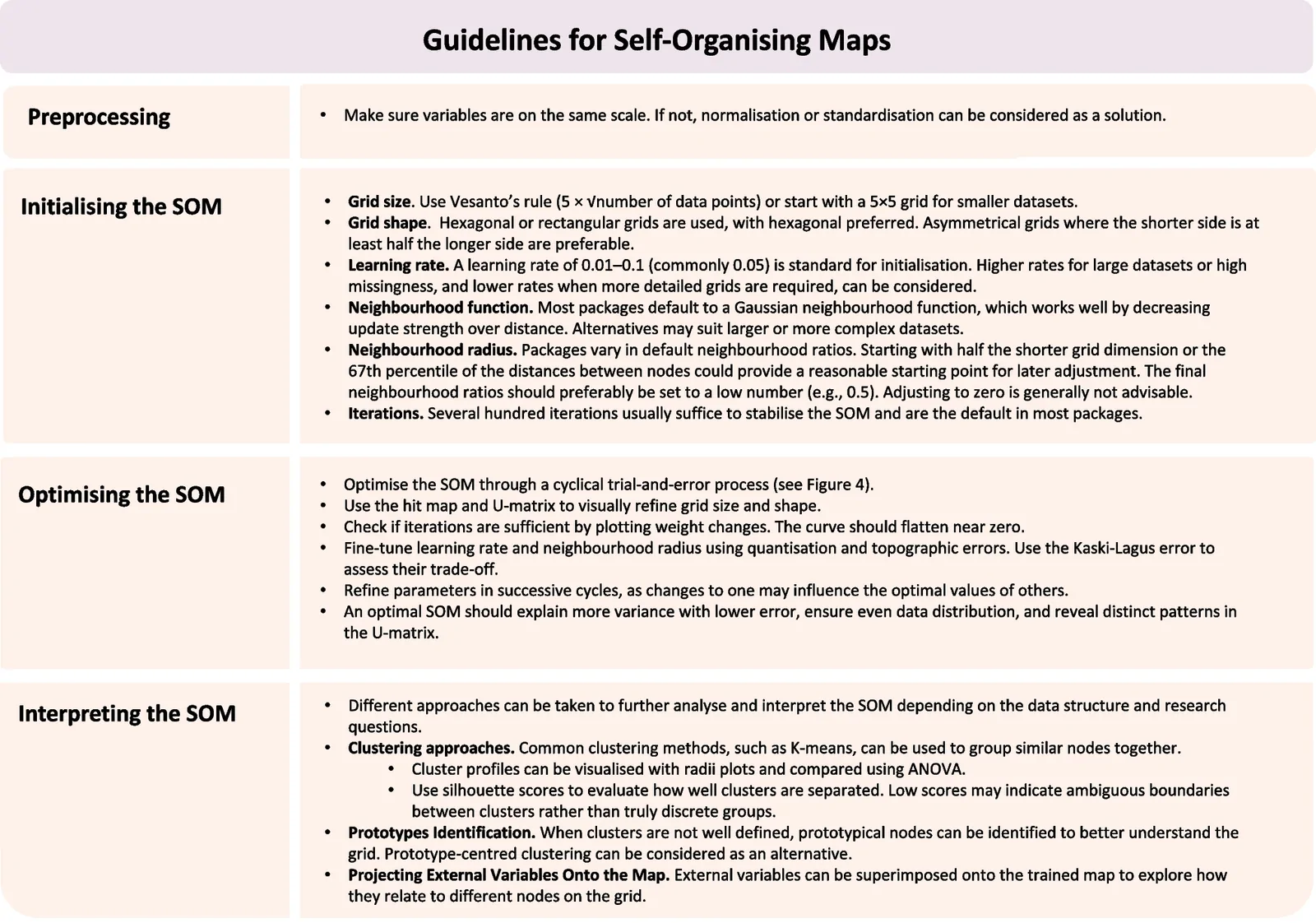
Summary of recommended guidelines for SOM

Building a SOM involves three main steps (following data preprocessing), namely initialisation, optimisation, and interpretation (Fig. 2B). First, an initial map is set up with rough parameters. These are then fine-tuned by adjusting the grid size, shape, number of iterations, and other settings. These steps require careful decision-making to determine how detailed the final map should be, depending on the research questions and the dataset, allowing researchers to reveal either broader patterns or fine-grained heterogeneity. Once an optimal map is selected, the final stage is interpretation. While this can be accomplished through multiple approaches, this tutorial focuses on clustering similar nodes into distinct profiles. Below, we provide a step-by-step guide and introduce key terms for informed decision-making throughout the process. A flowchart summarising the guidelines is provided in Fig. 3.

### Data preprocessing

Before generating the SOM, data should be prepared to ensure all variables are on the same scale. Variables with larger ranges can disproportionately influence the results. For example, if symptom severity is measured on a 0–3 scale, while intelligence quotient (IQ) is measured on a standard score scale, IQ could dominate the learning process due to its larger range. Common approaches, such as normalisation, where each variable is rescaled to range between 0 and 1, can address this.

### Initialising the SOM

#### Grid features

##### Grid size

Determining the grid size by choosing the number of nodes is one of the most critical decisions, directly affecting the SOM quality. The grid is typically two-dimensional (e.g., 10 × 10), with larger and more complicated datasets requiring larger maps. A common heuristic recommends that the number of nodes should be approximately five times the square root of the number of data points (Vesanto et al., 2000). For example, for 100 participants, the map would include approximately 50 (5 × √100) nodes (e.g., 7 × 7 grid). For smaller datasets, researchers suggest starting with a small grid (e.g., 5 × 5) and gradually increasing its size while monitoring the quality of the map (Riese et al., 2020).

##### Grid shape

The grid shape also affects the quality of the SOM. The most common shapes are rectangular and hexagonal (although other topologies exist; López-Rubio & Díaz Ramos, 2014). While rectangular grids may be more computationally efficient, hexagonal grids are often preferred because neighbouring nodes are arranged more evenly around this shape, producing smoother grids that better preserve the topology of the data (Kohonen, 2013; Villmann, 1999).

Regardless of the shape, asymmetrical grids (e.g., 9 × 11 rather than 10 × 10), tend to improve cluster separation (Kohonen, 2001; Ultsch & Herrmann, 2005; Wendel & Buttenfield, 2010). It is also recommended that the shorter dimension be at least half the length of the longer side (Kohonen, 2001). This “one-half rule” reduces uneven updates at grid borders, where nodes have fewer neighbours.

#### SOM parameters

When constructing SOMs, several key parameters must be considered, as reviewed below.

##### Learning rate

The learning rate (*alpha*, ranging 0–1) controls how much the weights are adjusted during self-organisation. Higher values produce larger, faster updates, and lower values produce smaller, more gradual changes, affecting training speed and map stability. SOMs typically use a decreasing learning rate, starting high to allow large adjustments early in training when the structure is still forming, and then gradually declining for fine-tuning in later stages. The initial learning rate should be high enough to allow rapid adaptation, but not too high that it overshoots optimal weight values. The final learning rate should allow careful fine-tuning without disrupting the grid’s structure.

Many packages use an initial learning rate between 0.01 and 0.1 (Van Gassen et al., 2015; Wehrens & Kruisselbrink, 2023; Wittek et al., 2017). The *kohonen* package defaults to 0.05, which has been shown to be an efficient middle-ground that is neither overly liberal nor overly conservative (Arunachalam & Kumar, 2018; Tao et al., 2024; Wehrens & Buydens, 2007). Other packages, such as Python *miniSOM* (Vettigli, 2018), implement higher rates, although evidence comparing these defaults remains limited (Mersiovsky et al., 2018). Higher initial learning rates may be preferable for datasets with substantial proportions of missing values, especially when missingness is patterned rather than random (Khotimah et al., 2018), or for very large samples where faster training is required (Tao et al., 2024). Lower initial learning rates may be appropriate when finetuning is needed (Goodfellow et al., 2016). Another consideration is the decay algorithm used to reduce learning rate over time. The *kohonen* package applies a linear decrease in the learning rate to a default minimum of 0.01 (Wehrens & Kruisselbrink, 2023). Alternative decay functions are discussed elsewhere (Stefanovic & Kurasova, 2014).

##### Neighbourhood function and radius

As mentioned earlier, once the BMU is identified, surrounding nodes are also updated. The neighbourhood function determines the strength of these updates. The *kohonen* package supports Gaussian and bubble functions (Wehrens & Kruisselbrink, 2023), with the default Gaussian function applying stronger updates near the BMU and weaker updates to distant nodes. Gaussian function is generally suited for psychological data, but for large databases, alternatives may be considered (Clark et al., 2020; Natita et al., 2016; Ota et al., 2011; Stefanovic & Kurasova, 2014).

We also specify how far the influence of the BMU extends at each step, determined by the neighbourhood radius (*sigma*; Fig. 2B). Smaller values restrict updates to nodes near the BMU. Radii that are too small can reduce map quality by under-updating distant nodes, whereas overly large radii may reduce accuracy and increase error rates (Kolasa & Dlugosz, 2009). Default settings vary across packages (KratochvÌl et al., 2019; Vettigli, 2018; Wittek et al., 2017). The *kohonen* package uses the 67th percentile of the distances between nodes, which may be slightly large, but provides a reasonable starting point (Kolasa & Dlugosz, 2009). In practice, the “right” radius depends on the specific dataset and grid structure (Fukui & Numao, 2011), so trial-and-error should always be followed.

Like the learning rate, the neighbourhood radius decreases over time. A relatively low final radius (e.g., 0.5) is recommended to ensure only the closest nodes are updated in later stages (Bednar et al., 2004; Vesanto et al., 2003). It should be noted that some packages, including *kohonen* (Wehrens & Kruisselbrink, 2023), default to a final radius of 0, which may weaken local neighbourhood structure (Kohonen, 2013).

##### Number of iterations

The number of iterations also affects SOM quality. Too few iterations may lead to insufficient learning, where nodes fail to adequately capture the data structure, whereas too many may make the map too specific to the data, potentially reducing its generalisability. Most packages use a default of 100 to 500 iterations (Vettigli, 2018; Vialaneix et al., 2025; Wehrens & Kruisselbrink, 2023), but optimal values depend on the dataset and are best determined through trial-and-error.

### Optimising the SOM

Selecting an optimal set of parameters is highly dependent on the data and, therefore, involves a trial-and-error process guided by theoretical and empirical considerations. During this process, the quality of the SOM should be monitored as parameters are adjusted until a final set is chosen. Many parameter combinations may work well, and no single solution is strictly correct. It should also be noted that when the data contain a strong underlying structure, different reasonable combinations often affect fine details (e.g., exact node boundaries) rather than main patterns (Cottrell et al., 2001). Nonetheless, choices should be justified to ensure appropriate parameters are selected. Below, we outline key quality metrics for this process, followed by practical suggestions for parameter optimisation.

#### Quality and error terms

Three complementary error measures guide optimisation. Quantisation error quantifies how well the grid matches the actual data, measuring the average distance between each data point and its BMU. Lower values indicate a better match (with zero representing a perfect fit). Since quantisation error is expressed in the original data units, which often lack standardised norms, it is often interpreted alongside explained variance, which is the proportion of variability in the data captured by the SOM (analogous to intuitive units like *R*^2^ in regression). While no standard benchmark exists, higher explained variance generally indicates better performance. 

Low quantisation error does not necessarily indicate a well-organised map (Uriarte & Martín, 2008). Similar data points may still be assigned to distant nodes on the grid, even if each data point is near its BMU, a problem quantisation error cannot detect. The topographic error addresses this by measuring whether similar nodes are grouped together (Pölzlbauer, 2004). The topographic error estimates the proportion of data points for which the BMU and second-best-matching unit are *not* adjacent (Kohonen, 2001), with lower values indicating better topology.

Quantisation and topographic errors capture different aspects of SOM quality that can sometimes conflict. To solve this, Kaski and Lagus (1996) proposed a combined measure, balancing accuracy (how well the grid represents the data) and organisation (how well relationships are preserved across the grid). Lower Kaski–Lagus error indicates higher SOM quality. The error metrics depend on the data, so there are no fixed thresholds for what constitutes “low” error. However, they are useful for comparing SOMs trained on the same dataset, and can guide the fine-tuning of the learning rate, neighbourhood function, and number of iterations required (Wendel & Buttenfield, 2010).

#### Refining parameters

As noted, identifying an optimal SOM involves trial and error, where different maps are tested and compared (Kohonen, 2001, 2013). The aim is to end up with a SOM with relatively low error, where data points are accurately represented on the grid and similar nodes are grouped together. This can be evaluated using the quality metrics alongside visually inspecting the maps.

**Fig. 4:**
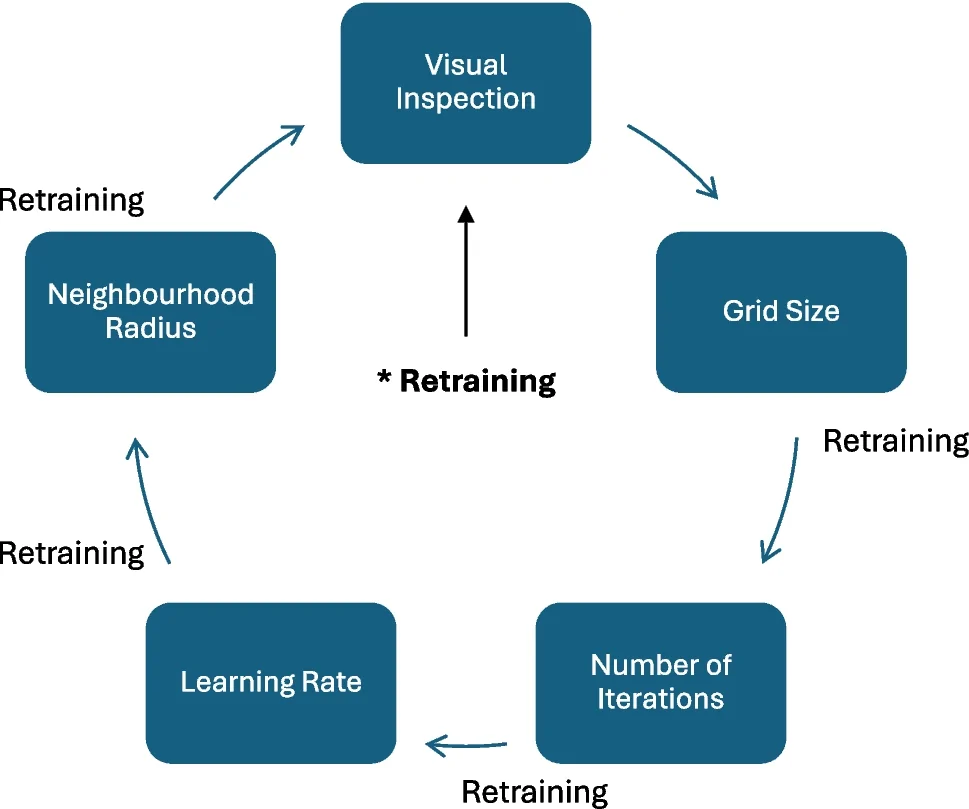
Cyclical trial-and-error approach to SOM optimisation. *Note*. A schematic illustration of the SOM optimisation process. This is a cyclical trial-and-error procedure aimed at refining the SOM’s structure and performance. We recommend beginning with a visual inspection of the hit map and U-matrix to assess data distribution and separation. Based on these insights, adjust the parameters as needed. After each tuning, retrain the SOM and repeat the cycle until the map quality is satisfactory

We recommend beginning with the grid size and shape, as these strongly affect all other parameters (Gonzalez et al., 1997), and then adjusting the iterations, learning rate, and neighbourhood radius through a cyclical process (Fig. 4). A fully worked example is included in the accompanying R code.

**Fig. 5:**
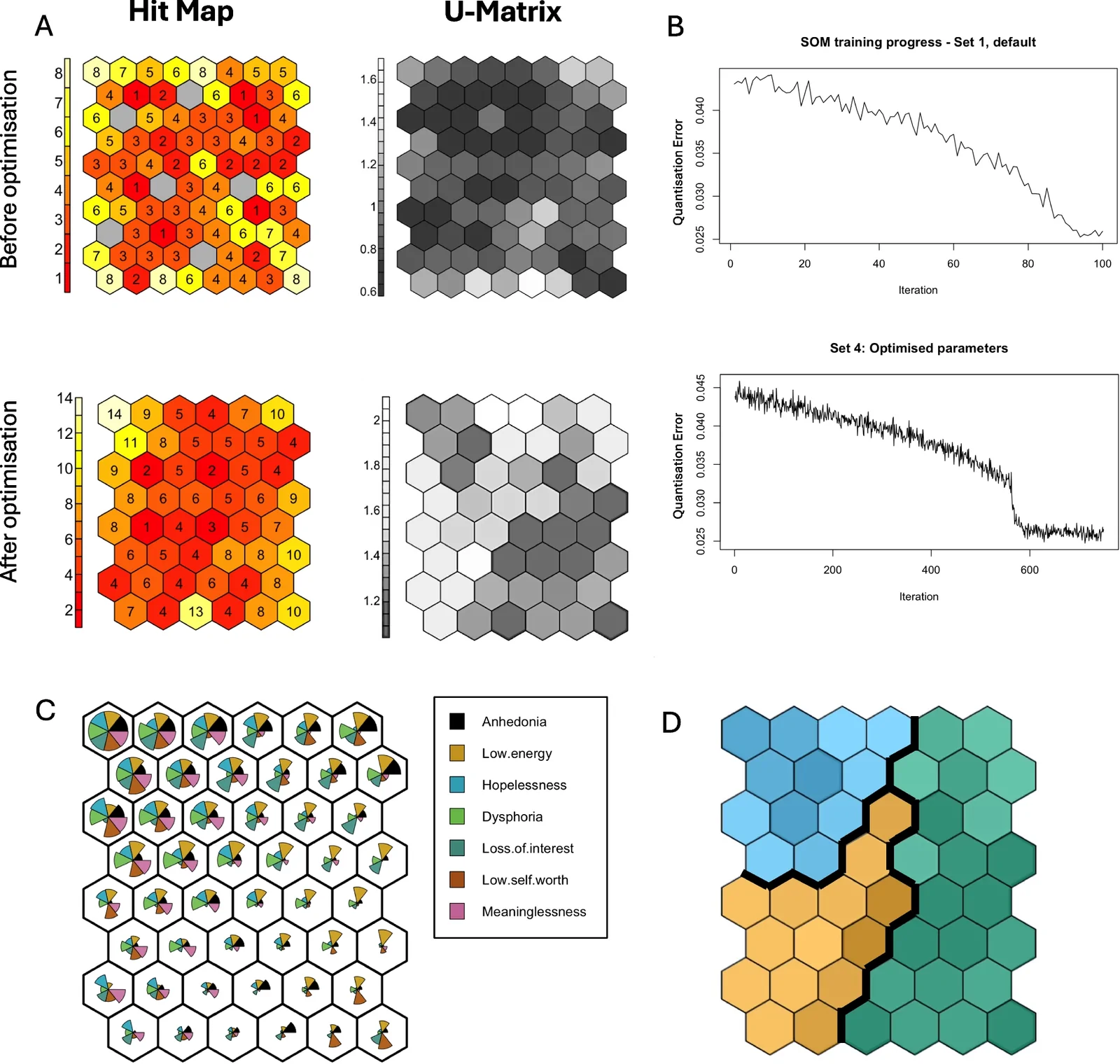
SOM optimisation and clustering. *Note*. **A** The two diagnostic tools for evaluating SOM quality and structure. The hit map (left panels) shows how the 301 participants are distributed across the hexagonal grid nodes. Before optimisation, the distribution appears more uneven, with some nodes having high hit counts (in yellow) and others remaining empty (in grey). The U-matrix reveals the underlying structure of depression subtypes. Before optimisation, the distance patterns are less coherent, making it difficult to identify clear cluster boundaries. After optimisation, the U-matrix shows clearer patterns where lighter regions indicate boundaries between different depression phenotypes (e.g., separating individuals showing more cognitive symptoms like hopelessness than individuals showing more somatic symptoms like anhedonia), while darker regions represent homogeneous areas where participants share similar symptom profiles. **B** These graphs track the quantisation error over training iterations. In the top graph, the relatively short training (100 iterations) does not fully optimise the SOM's ability to capture data patterns, with the error still dropping until the final few iterations. In the bottom panel (750 iterations), quantisation error declines more steeply until it stabilises well before training ends. **C** The figure displays the average symptom severity for participants assigned to each node on the optimised SOM grid. Each hexagon represents a node on the grid, with the pie chart inside showing the relative magnitude of each of the seven depression symptoms. **D** Displays a three-cluster solution that was found to fit the data. Each node is coloured according to its cluster assignment, with thick lines indicating the boundaries between clusters

Choosing the optimal grid size depends primarily on the data, but standard error metrics are insufficient for this purpose. As the number of nodes increases, the average distance between data points and their BMU decreases mechanically, so quantisation error and explained variance cannot distinguish better-fitting maps from larger ones (de Bodt et al., 2002; Pölzlbauer, 2004). Similarly, topographic error is also limited for this purpose (Pölzlbauer, 2004). Instead, two visual diagnostics are recommended to guide grid size selection (Fig. 5A).

The first, the hit map, displays the number of data points assigned to each node. Ideally, data points should be relatively evenly spread. Many empty nodes may indicate that the grid is too large, while numerous highly concentrated nodes indicate it is too small or poorly shaped. The second diagnostic map, the unified distance matrix (U-matrix), shows how similar neighbouring nodes are, with lower values indicating greater similarity. When distinct profiles exist, they appear as small “islands” of similar nodes separated by higher-value boundary regions. Uniformly low values across the U-matrix indicate absence of distinct clusters or an undersized grid. Excessive gaps suggest that the grid is too large, making structure detection difficult. Together, these diagnostic maps guide optimisation of the grid size.

Once grid size is selected, the next step is assessing whether the SOM has trained for enough iterations by plotting the total change in node weights over time (Fig. 5B). A curve flattening near zero indicates the map has stabilised, and further iterations are unlikely to yield improvements. Next, error metrics can guide fine-tuning of the learning rate and neighbourhood radius. In our case, we tested a range of values and selected appropriate settings based on error metrics and map visualisations (Table 2).

**Table 2:** Example of SOM parameter settings and error metrics during optimisation

Parameter set	QE	Explained variance	TE	Kaski–Lagus error	Notes
**Set 1** Grid size: 8 × 10 Learning rate = 0.05, 0.01 Neighbourhood radius = 5.29 Iterations: 100	1.36	76.25%	0.12	2.23	Grid size 8 × 10 too large (many empty nodes in hit-map). Reduced grid size is needed
**Set 2** Grid size: 6 × 8 Learning rate = 0.05, 0.01 Neighbourhood radius = 4 Iterations: 100	1.58	72.43%	0.16	2.64	Improved topography with 6 × 8 grid, but QE did not stabilise. Increased number of iterations is needed
**Set 3** Grid size: 6 × 8 Learning rate = 0.05, 0.01 Neighbourhood radius = 4 Iterations: 750	1.45	74.68%	0.16	2.68	QE stabilised at 750 iterations. All error metrics improved from Set 2
**Set 4** Grid size: 6 × 8 Learning rate = 0.08, 0.01 Neighbourhood radius = 4 Iterations: 750	1.44	74.88%	0.11	2.51	Optimal SOM selected. Increased learning rate to 0.08, which reduced QE, TE, and Kaski–Lagus error while maintaining topological quality. Neighbourhood radius was retained from Set 3 as it produced optimal topography

*QE* quantisation error; *TE* topographic error

Since SOM parameters are interdependent (Kohonen, 2001), several rounds of refinement are often needed. In our example, the final model explained an additional 2.5% of variance, had a more even distribution of data points, and a U-matrix highlighting distinct patterns (Fig. 5A). For transparency and replicability, we recommend documenting all decisions throughout the process, including initial and final SOM configurations. Including a summary table listing each SOM setup alongside its error metrics is recommended (Table 2).

### Assessing the reliability of the SOM

Once a final SOM has been selected, we advise assessing its reliability. This applies to all data-driven approaches and is not unique to SOMs (Takeuchi, 2025). While a comprehensive review of machine learning validation methods is beyond the scope of the paper and is available elsewhere (Pargent et al., 2023; Takeuchi, 2025), we outline a practical approach below, accompanied by an R code example in the Supplementary Materials.

Similar to other machine-learning approaches, training a SOM involves a degree of randomness (e.g., random starting weights, or the order in which participants are presented), so results can vary slightly each time the model is run. Therefore, it is important to assess its reliability to ensure that the patterns identified by the SOM are meaningful and not simply due to chance. One way to do this involves training the SOM on a randomly selected subset of the data rather than the full dataset (e.g., around 70%, depending on the dataset size and number of variables). This process is then repeated multiple times, each time using a different random subset. By comparing the results across these subsets, we can evaluate whether the SOM produces consistent patterns. If the main structures remain similar across runs, the SOM can be considered reliable, suggesting that its patterns reflect the data rather than chance variation in training.

### From maps to meaning: Further analysis of SOMs

Once a reliable SOM is selected, we can explore the patterns it reveals. In Fig. 5C, each node displays the average values of variables for the participants assigned to it. In our example, the grid comprised 48 nodes, each representing a small group of participants with highly similar symptom combinations. These should not be interpreted as 48 distinct “types” of depression. Instead, they provide a fine-grained tiling of the symptom landscape, where neighbouring nodes represent closely related profiles and distant ones represent divergent patterns, forming a continuum.

To further make sense of the SOM, different analytical approaches can be applied depending on the data and research questions. Further clustering the nodes is useful when broad groups are needed or when clear clusters emerge. Node-level information can also be used to address a wide range of questions, such as how variables vary across the map, how profiles change along gradients, and how regions on the map correspond to external variables. In this paper, we focus on clustering, one of the most widely used methods combined with SOMs (Miljković, 2017; Vesanto & Alhoniemi, 2000), and provide practical guidance on its implementation. We then provide a brief overview of alternatives.

#### Clustering the SOM

Since neighbouring nodes learn relatively similar patterns, researchers can determine whether they are similar enough to be grouped together. This enables the identification of groups of individuals who share common profiles (e.g., symptom profiles; Mareva et al., 2024). To do this, researchers can apply an additional clustering step (Kohonen, 1990; Vesanto & Alhoniemi, 2000).

A common approach is to apply *k*-means clustering, which partitions the nodes into a predefined number of clusters (*k*). After clustering, the grid should be inspected to ensure clusters form coherent and contiguous regions (Fig. 5D). If nodes from the same cluster are scattered across the grid, it may indicate a clustering issue.

With clearly defined clusters, simple methods (e.g., analysis of variance [ANOVA]) can be employed to visualise and compare them (Astle et al., 2019; Mareva et al., 2024). In our example (Fig. 1), three distinct depression clusters were revealed. The first (blue) represents severe multidimensional depression, with elevated symptoms across all domains. In the second (green) cluster, individuals primarily show fatigue and anhedonia with relatively lower cognitive-emotional symptoms, resembling a somatic manifestation of depression, which has previously been associated with a chronic course (Billones et al., 2020). The third (orange) cluster reflects a pattern marked by hopelessness and meaninglessness alongside dysphoria but lower somatic symptoms. This combination warrants careful clinical attention, as these features, together with relatively preserved energy, have been linked to increased suicide risk (Allen, 2022; Ribeiro et al., 2018; Rogers et al., 2018).

Comparing these clusters with the results of other common methods applied to the same data illustrates what SOMs add (see Supplementary Text). EFA identified two symptom dimensions that broadly mirror the symptoms distinguishing the second and third clusters, but does not identify distinct groups of individuals. Individuals can subsequently be grouped using their factor scores (e.g., Drossel et al., 2023), but these groups are then defined by the underlying dimension rather than the original symptoms, and different combinations of symptoms may receive similar scores. Network analysis shows how symptoms relate to one another, but not how symptom patterns differ across individuals. Finally, PCA with *k*-means clustering and latent profile analysis (LPA) identified profiles that differed primarily in overall symptom severity rather than in specific symptom patterns.

Importantly, while *k*-means produces discrete clusters, nodes (and, by extension, individuals assigned to them) located near cluster boundaries share characteristics with adjacent clusters. This creates gradual transitions between clusters rather than sharp divides, allowing SOMs to move beyond rigid categorical classification that has historically constrained psychological research (Dalgleish et al., 2020). At the same time, we can also evaluate how distinct or overlapping the clusters are.

One way to do that is to assess how similar nodes within each cluster are, and how different they are from nodes in other clusters. This can be quantified using *silhouette scores*, an index ranging from − 1 to + 1, with values above 0.5 typically indicating well-defined clusters (Kaufman & Rousseeuw, 1990; Rousseeuw, 1987).

Beyond how well separated clusters are, it is also important to assess cluster stability, that is, whether the same participants are grouped together when the analysis is repeated. One way to test the stability of SOM clusters is using a bootstrap approach, a common method in clustering validation (e.g., Binhimd & Kalantan, 2023; Yu et al., 2019). This approach repeatedly draws samples from the full dataset (e.g., hundreds of times), retrains the SOM, and repeats the clustering on each sample. For each cluster, stability is quantified using the *Jaccard Index*, calculated as the number of participants shared between the original cluster and its matching cluster, relative to the total number of participants in either cluster (Hennig, 2008). This index reflects how reliably a cluster is reproduced across resampled datasets, with values below 0.5 indicating poor stability, while values above 0.75 indicate good stability.

It is important to note that cluster labels are arbitrary, and the map’s layout can change each time the SOM is retrained. For example, a profile could be labelled Cluster A and occupy the top left of the map in one run, yet be labelled Cluster C and occupy the bottom right in another, while containing the same participants. To account for this label switching, we suggest using the Hungarian method (Kuhn, 1955) to match the new clusters to the original ones based on the greatest participant overlap (see Supplementary Materials code).

#### Alternative approaches

While combining clustering with SOMs is common (Miljković, 2017), alternative methods may be useful, particularly when clustering performs poorly. In our data, a silhouette score of 0.21 (see Code) indicated that profiles did not form clearly distinct categories. This is unsurprising since discrete categories often fail to describe psychological constructs (De Boeck et al., 2023; Linden & Hönekopp, 2021). Consequently, low silhouette scores are common in psychological research (Ghosh et al., 2022; Rennie et al., 2020), including studies identifying depression subgroups (Eder et al., 2025; Hannon et al., 2025; Xu et al., 2025). Such results may reflect genuine properties of the data, rather than a methodological limitation. In these cases, researchers can use the resulting SOM to further investigate the data. Although this paper focused on SOM clustering, we wish to outline two alternative ways to analyse SOMs.

##### Using prototypes to interpret clusters

When silhouette scores are low, nodes near cluster boundaries resemble those of the adjacent cluster, making average cluster profiles potentially misleading. Instead, it may be more informative to focus on the most representative node within each cluster (e.g., the one minimising the average distance to all other nodes in that cluster), treating it as the cluster prototype (Gao et al., 2023; Semoglou et al., 2025). This approach reveals distinct cluster cores while acknowledging the continuous gradient of profiles between prototypes (Fig. 1).

##### Interpreting SOMs using external variables

Another option is to forgo clustering and instead project external variables of interest (e.g., diagnosis, treatment outcomes, risk markers) onto the map. This allows researchers to track how map regions relate to external variables, while maintaining the continuous nature of the map (Brereton, 2012; Ejarque-Gonzalez & Butturini, 2014). For example, diagnostic categories (e.g., major depressive disorder, persistent depressive disorder, bipolar disorder), clinician-rated assessments (e.g., suicide risk scores), or treatment outcomes can be superimposed onto the SOM to see whether they align with regions corresponding to specific profiles.

These methods are neither exclusive nor exhaustive and can be combined with other approaches depending on the research aims.

## Other advanced applications of SOMs

Multiple extensions of SOMs have been developed that may hold promise for psychological research. Although we do not aim to provide a comprehensive review of these extensions, we highlight selected developments and their potential applications.

### Supervised SOM

Traditional SOMs group individuals by similarity alone, making them useful for exploring patterns without prior knowledge guiding the process. In contrast, supervised SOMs incorporate known outcome information during map construction (Kohonen, 2001), allowing the map to organise individuals not only by similarity, but also by their relationship to a target outcome. For example, supervised SOMs were previously used to identify which acoustic features in speech best predicted emotional states (Tuckova et al., 2011).

### Growing hierarchical SOM

Hierarchical SOMs extend the standard SOM by arranging multiple grids hierarchically, capturing increasingly granular patterns (Lampinen & Oja, 1992). The first layer groups the entire dataset, and each group feeds into a SOM in the next layer to identify finer sub-patterns. Hierarchical SOMs are often combined with growing SOMs, which begin with a few nodes and expand automatically during training, eliminating the need to predefine the grid size (Alahakoon et al., 2000; Rauber et al., 2002).

In psychology, growing hierarchical SOMs were used to identify a three-layered structure of cognitive abilities and six higher-order cognitive profiles in children with learning and developmental disorders (Siugzdaite et al., 2020). Similarly, adolescent mental health data identified over 140 sub-profiles organised into eight higher-order profiles, ranging from flourishing to widespread difficulties (Bennett et al., 2026). Despite the hierarchical nature of many psychological constructs (Conway et al., 2019; Fajkowska, 2018; Schurz et al., 2021), growing hierarchical SOMs remain scarcely used in psychology, suggesting an untapped opportunity for future research.

### Temporal extensions of SOMs

SOMs can also help examine how individuals change over time. For example, they have been used to identify well-being profiles of older adults, showing that transitions between profiles over 2 years were linked to digital literacy (Cervantes et al., 2022). Several temporal SOM variants explicitly incorporate time (Barreto, 2007; Javed et al., 2024; Ouyang et al., 2024). One such advancement, the Self-Organized Multi-Modal Longitudinal Map (SOM2LM; Ouyang et al., 2024), was trained on structural magnetic resonance imaging (MRI) data to group individuals by Alzheimer’s disease stage, allowing researchers to track individual disease progression over time. These developments offer promising avenues for clinically meaningful and personalised applications.

## Limitations

Despite their strengths, potential limitations of SOMs should be considered. A key challenge is their sensitivity to parameter settings. Poorly chosen parameters can produce unstable or misleading maps. This is compounded by the lack of widely accepted best practices. We aimed to address this by providing research-informed recommendations for parameter selection suitable for psychological applications. However, while our guidelines are consistent with prior work (Kohonen, 2001; Licen et al., 2023; Wendel & Buttenfield, 2010), they may not generalise to all contexts.

The choice of clustering method (if applied) and its validation also warrants consideration. Although we highlighted one approach, other clustering algorithms could be used to achieve comparable goals. A full review is beyond the scope of this paper, but overviews are available elsewhere (Yin et al., 2024). As with other data-driven methods (Takeuchi, 2025), identified patterns should be checked for reliability and ideally validated on independent data (Chen & Wojcik, 2016).

The lack of formal methods for determining power or sample size in SOMs is another limitation. Published studies range from a few dozen participants (Almeida et al., 2024; Khosrowabadi et al., 2010; Yorek et al., 2016) to several hundred (Astle et al., 2019; Kristensen et al., 2024; Leachman et al., 2024), with little guidance on minimum sample sizes needed. Some evidence suggests SOMs may be more robust to smaller samples than standard clustering methods, provided that parameters are carefully chosen (Kiang et al., 2007), but further research is needed to establish this more firmly.

Finally, like all data-driven methods, SOMs are constrained by the quality of the data they use (Browning et al., 2020). Measurements that are atheoretical or imprecise cannot be rescued by any analysis. This principle applies across psychological and cognitive sciences, neuroscience, and computational psychiatry, where the goal is to identify meaningful individual differences. Combining theory-driven with data-driven approaches has been proposed as a particularly promising strategy to address this challenge (Huys et al., 2016; Karvelis et al., 2022). Although such integration is possible with SOMs, it has yet to be widely applied to SOMs in psychological research and represents a promising avenue for future research.

## Conclusions

This paper presented a practical tutorial for applying SOMs to psychological research, alongside key considerations and recommendations to support robust and transparent applications. SOMs offer a powerful approach for analysing psychological phenomena, making nebulous and poorly understood constructs more tractable and interpretable. As the field moves towards richer, more complex datasets, SOMs offer a promising tool for discovering meaningful structures without oversimplification. With careful implementation, clear reporting, and appropriate validation, SOMs can support a more nuanced understanding of individual differences, development, and clinical phenomena in psychological science.

## Supplementary Information

Below is the link to the electronic supplementary material.

Supplementary file1 (PDF 2606 kb)

## Data Availability

All materials for this manuscript, including the analysis script, RMarkdown file, and data, are openly available in the following OSF repository: https://osf.io/8znkm/overview?view_only=9801ece30ae9422a81d213487d6c6b5f.

## Footnotes

1 Other person-centred alternatives, such as latent class analysis, are also available, but they require relatively large samples for stable solutions, rely on stronger assumptions about the data, and still force individuals into a small number of discrete categories (Sinha et al., 2021).

2 The sample comprised adults recruited in Israel via social media and snowball sampling (Age_mean_ = 31.5 ± 9.5; 84% female). All participants provided ethics-approved informed consent (Tel Aviv University, 0004487–3). The data in this study were used as an illustrative case and were not pre-registered. All analyses were conducted in R v4.4.1 (R Core Team, 2021). SOMs were trained using the kohonen v3.0.12 package (Wehrens & Kruisselbrink, 2023) and further analysed and visualised with aweSOM v1.3 (Boelaert et al., 2022). Additional visualisations were produced using ggplot2 v3.5.1 (Wilkinson, 2011). *K*-means clustering was carried out using the basic stats package, and silhouette scores were computed with the cluster v2.1.8.1 package (Maechler et al., 2021).

3 In this tutorial, we focus on an intuitive non-technical explanation of the SOM algorithm, aiming to make the method accessible to researchers without a strong computational background. For readers interested in formal derivations and technical detail, a brief and accessible overview of SOMs fundamentals is provided elsewhere (Miljković, 2017). We also strongly recommend Kohonen’s (2013) practical summary of the core algorithm and its parameters, alongside his seminal book presenting a comprehensive description of the method (Kohonen, 2001). Further development of the mathematical foundations of SOMs is presented in a more technical account (Yin, 2008).

## Declarations

### Prior versions

A preprint of this paper is available at https://doi.org/10.31234/osf.io/dy6z2_v3.

## Glossary

Best Matching Unit (BMU): The “winning” position on the map that is most similar to a specific data point (e.g., can be a set of variables from one participant).
Data points: Individual observations from the dataset that are used to train the SOM. In psychological research, this is typically the participants.
Grid: The two-dimensional layout of the Self-Organising Map (SOM), where each position reflects a different type of data pattern.
Hit map: A visual display showing how many data points are assigned to each position on the map. It helps identify dense and sparse areas in the data.
Iterations: The number of times the SOM goes through the entire dataset during training.
Kaski-Lagus error: A combined measure that accounts for both the accuracy of how well the map represents the data and the preservation of its topological structure. Lower values indicate a map that balances data representation with smooth organisation.
Learning rate (α): A value that determines how quickly the map updates as it adjusts to the information it receives. A higher value means bigger changes, a lower value means smaller, more gradual changes.
Neighbourhood function: A rule that determines how strongly nearby positions on the map are updated when the map learns from a data point. The influence usually decreases with distance from the best matching unit.
Neighbourhood radius (σ): The size of the area around the best matching unit that is affected during each update in training.
Node: A position on the map that represents a summary of similar data points. Each node holds average values that reflect the characteristics of the data assigned to it.
Quantisation error: A measure of how well the map represents the original data. Lower values mean better representation.
Topographic error: A measure of how well the map keeps similar data points close to each other. It checks how often the two best-matching positions for a data point are not next to each other on the map. Lower values mean better preservation of the data structure.
U-Matrix (Unified Distance Matrix): A visualisation that shows the distances between neighbouring map positions. Larger distances (shown as higher values) suggest clearer boundaries between different patterns in the data.
