# Supplementary material for: A practical guide to self-organising maps in psychological research
Source: Behav Res Methods. 2026 Nov 12;58(12):350. doi: 10.3758/s13428-026-03198-4 (PMC13759590; doi:10.3758/s13428-026-03198-4)
Supplement: Supplementary file 1 [file 13428_2026_3198_MOESM1_ESM.pdf]

title: “A Practical Guide to Self-Organising Maps in Psychological Research” authors: “Shalev I., Trachtenberg, E., Jelen, M. B., Mils, W., Bennett, M., Lawson, R. P., Dalgleish, T. & Astle D. E.” date: “2025-09-15” output: word_document

```
# Load Required Libraries
```

```
#

# Data Manipulation
library(dplyr)
```

```
##
```

```
## Attaching package: 'dplyr'
```

```
## The following objects are masked from 'package:stats':
```

```
##
##     filter, lag
```

```
## The following objects are masked from 'package:base':
```

```
##
##     intersect, setdiff, setequal, union
```

```
# Modeling and Statistics
```

```
library(kohonen)
library(cluster)

# Visualisation
library(ggplot2)
library(aweSOM)
library(ggpubr)
library(fmsb)
library(reactable)
library(RColorBrewer)

# Load Data
#

setwd("~/Desktop/Research/postdoc/For paper with Estherina/SOM Paper/Most recent/code")
data_som_raw <- read.csv("SOM_Data.csv", stringsAsFactors = FALSE)
```

```
# The data was drawn from Trachtenberg et al. (2024) consisting of 301 participants scoring mild or higher on the DASS Depression subscale. We examined seven items of the scale, reflecting core dimensions of depression including anhedonia, fatigue, hopelessness, dysphoria, loss of interest, low self-worth, and meaninglessness.

# ==================================================================
#              ------  1. Data Preprocessing -----
# ==================================================================

# 1.1. Clean and Format Data
# ──────────────────────────────────────────────────────────────────
# Keep the ID column (first column) for reference, but convert all other item columns to numeric. suppressWarnings handles potential non-numeric characters (becoming NA).

data_som_num <- data_som_raw %>%
  mutate(across(-1, ~ suppressWarnings(as.numeric(.))))

# 1.2. Scaling Decision
# ──────────────────────────────────────────────────────────────────
# WHY RESCALE? Some datasets have variables on very different scales (e.g., age in years vs. annual income in dollars). In such cases, rescaling ensures that variables with larger ranges don't dominate the SOM training.

# DECISION: All DASS depression items are already on the same 0–3 Likert scale. We maintain the original scaling for better interpretability.

do_scale <- FALSE # Set to TRUE to force 0-1 scaling

if (isTRUE(do_scale)) {
  normalize_01 <- function(x) {
    if (all(is.na(x))) return(x)
    return((x - min(x, na.rm = TRUE)) / (max(x, na.rm = TRUE) - min(x, na.rm = TRUE)))
  }
data_som_num <- data_som_num %>%
mutate(across(-1, normalize_01))
  }

# 1.3. Convert to Matrix
# ──────────────────────────────────────────────────────────────────
```

```
# The kohonen package requires data to be a numeric matrix.
# We also remove the ID column, as it is not a feature used for training the map.

data_som <- data_som_num %>%
  dplyr::select(-1) %>%
  as.matrix()

# Note: the data should be numeric and in a matrix format
```

Self-Organising Map (SOM): Setup, Training, Evaluation, and Visualisation

This script sets up, trains, and evaluates multiple SOM configurations, and visualises node counts and distances.

```
# ==============================================================
#             ------  2. Initialising the SOM  -----
# ==============================================================

# 2.1. Determine the Grid Size
#

# Vesanto's rule (Vesanto, 2000): total number of nodes (total_nodes) ≈ 5 × √N, where N is the number of participants.

n_samples <- nrow(data_som)
total_nodes <- round(5 * sqrt(n_samples))

# Generate a symmetrical grid as a baseline
grid_side <- round(sqrt(total_nodes))

# Calculate an asymmetrical grid following the One-Half rule (Kohonen, 2001). The shorter side should be at least half the length of the longer side. Targeting a ratio where the shorter side is roughly 80% of the longer side.

side_short <- round(sqrt(total_nodes * 0.8)) # shorter side
```

```
side_long <- round(sqrt(total_nodes/0.8)) # longer side

cat("Suggested asymmetrical grid size:", side_short, "x", side_long, "\n")
```

```
## Suggested asymmetrical grid size: 8 x 10
```

```
# 2.2. Define the SOM Grid
# ——————————————————————————————————————————

som_grid <- somgrid(
  xdim = side_short,
  ydim = side_long,
  topo = "hexagonal", # Topology: "hexagonal" is preferred over "rectangular"
  neighbourhood.fct = "gaussian") # Gaussian neighbourhoods yield smoother, more stable training

# 2.3. Train Initial SOM
# ——————————————————————————————————————————
# We train a baseline model (Set 1) using default parameters to check basic convergence before optimisation.

set.seed(201); som_set1 <- som(
  data_som,
  grid = som_grid,
  alpha = c(0.05, 0.01), # default learning rate
  rlen = 100) # default number of iterations
```

```
# ==============================================================
#             ------  3. Optimising the SOM  -----
# ==============================================================

## 3.1. Visually inspect the SOM
# ——————————————————————————————————————————
# Before optimising hyperparameters (iterations, learning rate, etc.), we visually assess the structural quality of the initial map.

# Hit Map
# Purpose: Shows how many participants (samples) are classified into each node.
# What to look for:
```

```
# - Ideally, samples are spread relatively evenly across the map.
# - Watch out for too many "empty" nodes (grid might be too large).
# - Watch out for "overloaded" nodes containing huge chunks of data (grid might be too small).

plot(som_set1, type = "counts", main = "Hit map - Set 1, default", palette.name = heat.colors)
```

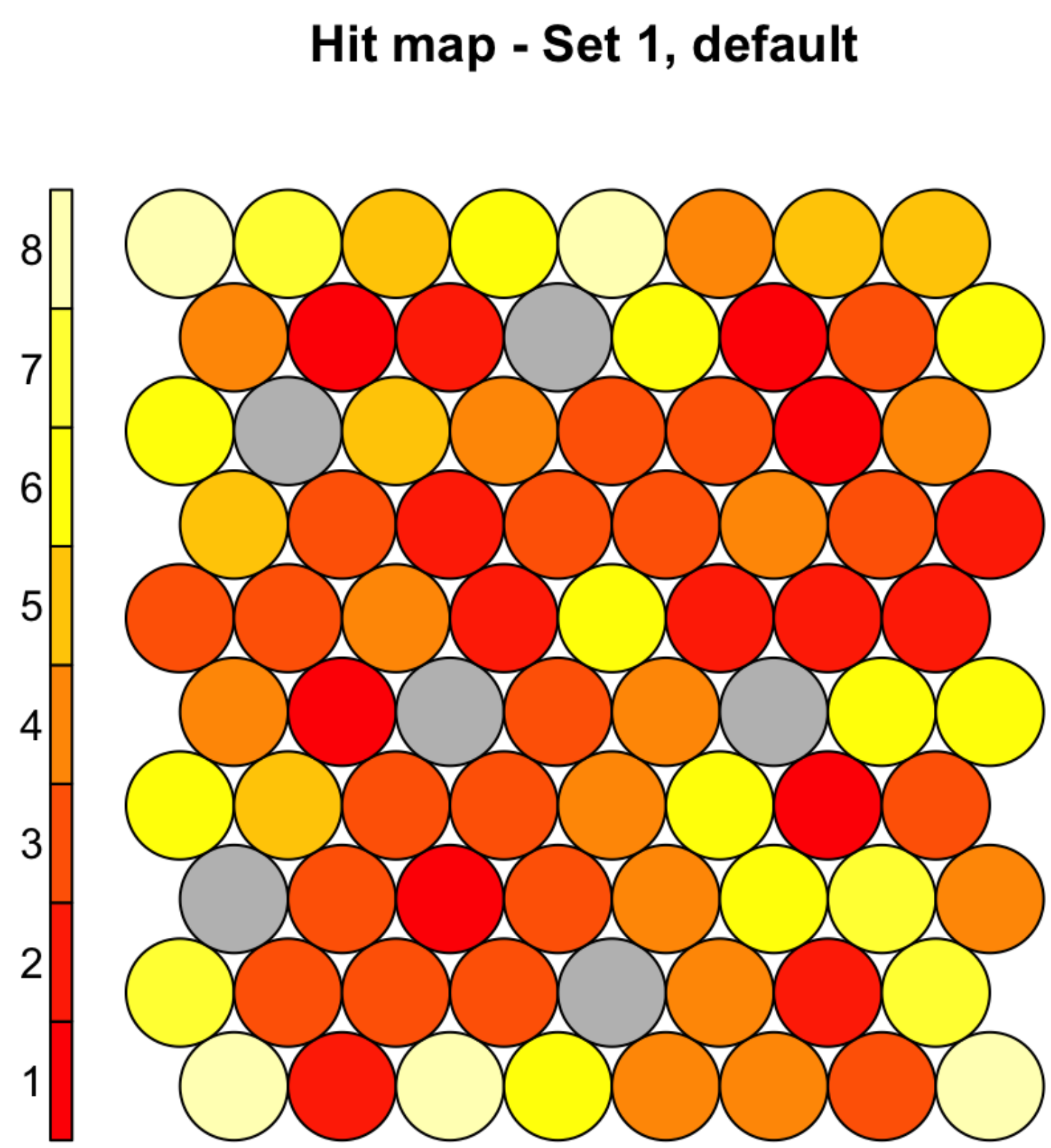


```
# U-Matrix (Neighbour Distances)
```

```
# Purpose: Visualises the distance between each node and its neighbors.
# Interpretation:
# - Dark/Red areas: Low distance (nodes are similar) -> Potential clusters.
# - Light areas: High distance (nodes are different) -> Boundaries between clusters.

plot(som_set1, type = "dist.neighbours", main = "U-Matrix - Set 1, default", palette.name = heat.colors) # Plot U-Matrix
```

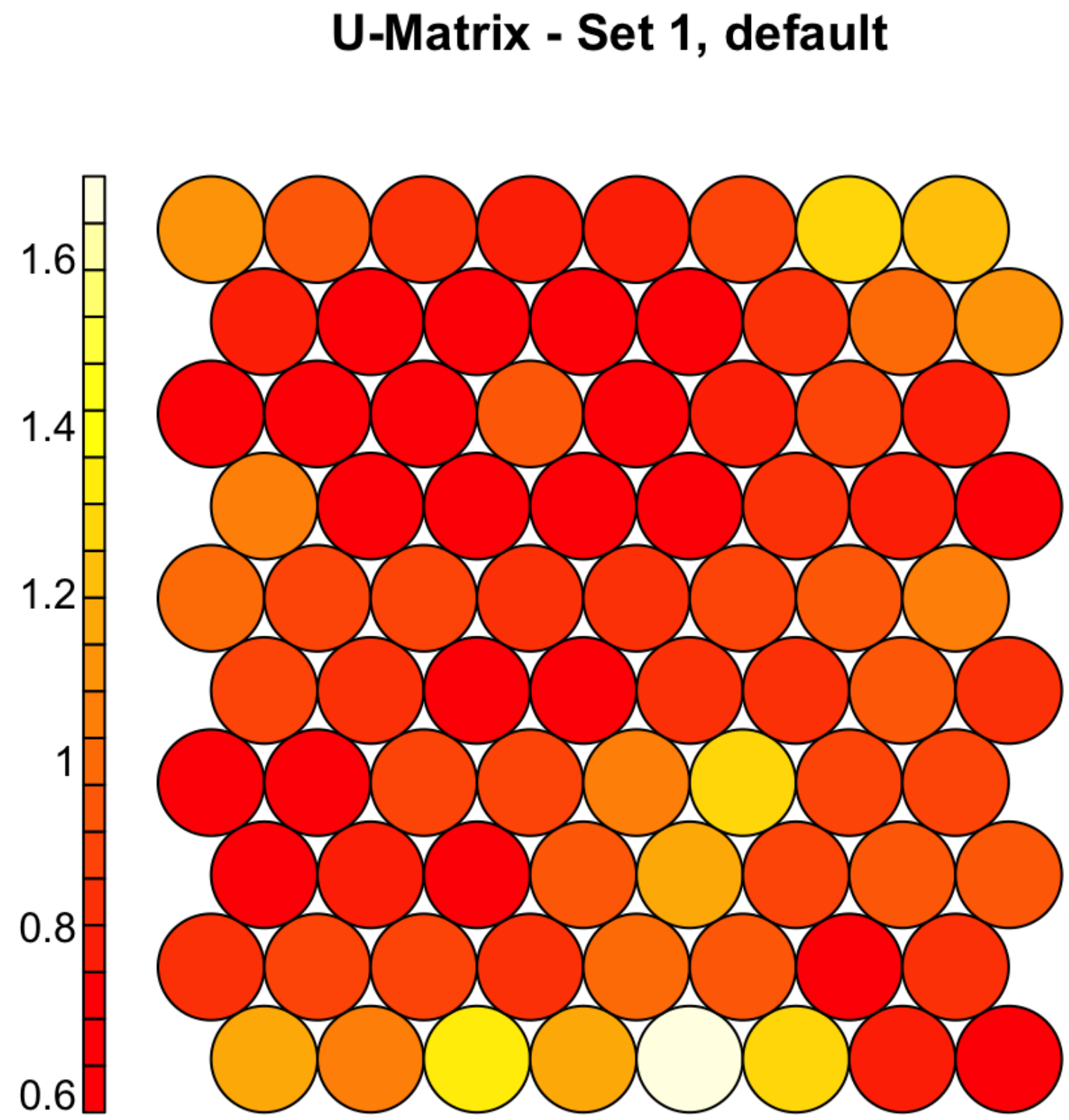


```
# ASSESSMENT NOTE:
```

The initial hit map shows many empty nodes (in grey). This may suggest that the grid is somewhat too large for the sample size.

```
## 3.1.1. Changing grid size (if necessary)
```

#

In practice, it is often preferable to have fewer empty nodes and slightly higher average hits per node, while still preserving enough resolution to reveal structure. We therefore also experiment with a smaller grid ultimately choosing a 6x8 configuration.

```
side_short <- 6
side_long <- 8

som_grid <- somgrid(
  xdim = side_short,
  ydim = side_long,
  topo = "hexagonal", # Topology: "hexagonal" is preferred over "rectangular"
```

```
  neighbourhood.fct = "gaussian") # Gaussian neighbourhoods yield smoother, more stable
training

set.seed(201); som_set1 <- som(
  data_som,
  grid = som_grid,
  alpha = c(0.05, 0.01), # default learning rate
  rlen = 100) # default number of iterations

plot(som_set1, type = "counts", main = "Hit map - Set 1, default", palette.name =
heat.colors)
```

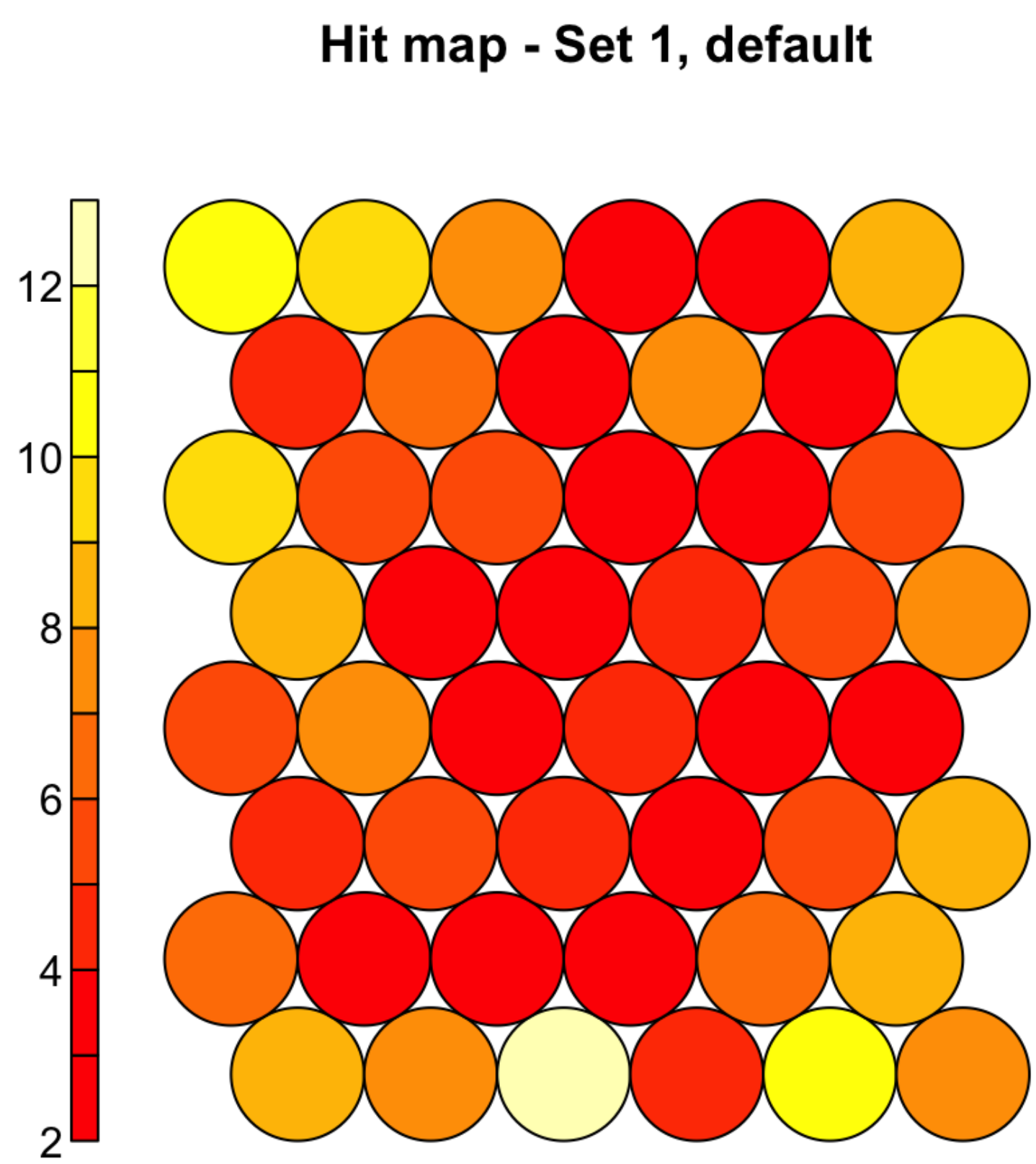


```
plot(som_set1, type = "dist.neighbours", main = "U-Matrix - Set 1,
default", palette.name = heat.colors) # Plot U-Matrix
```

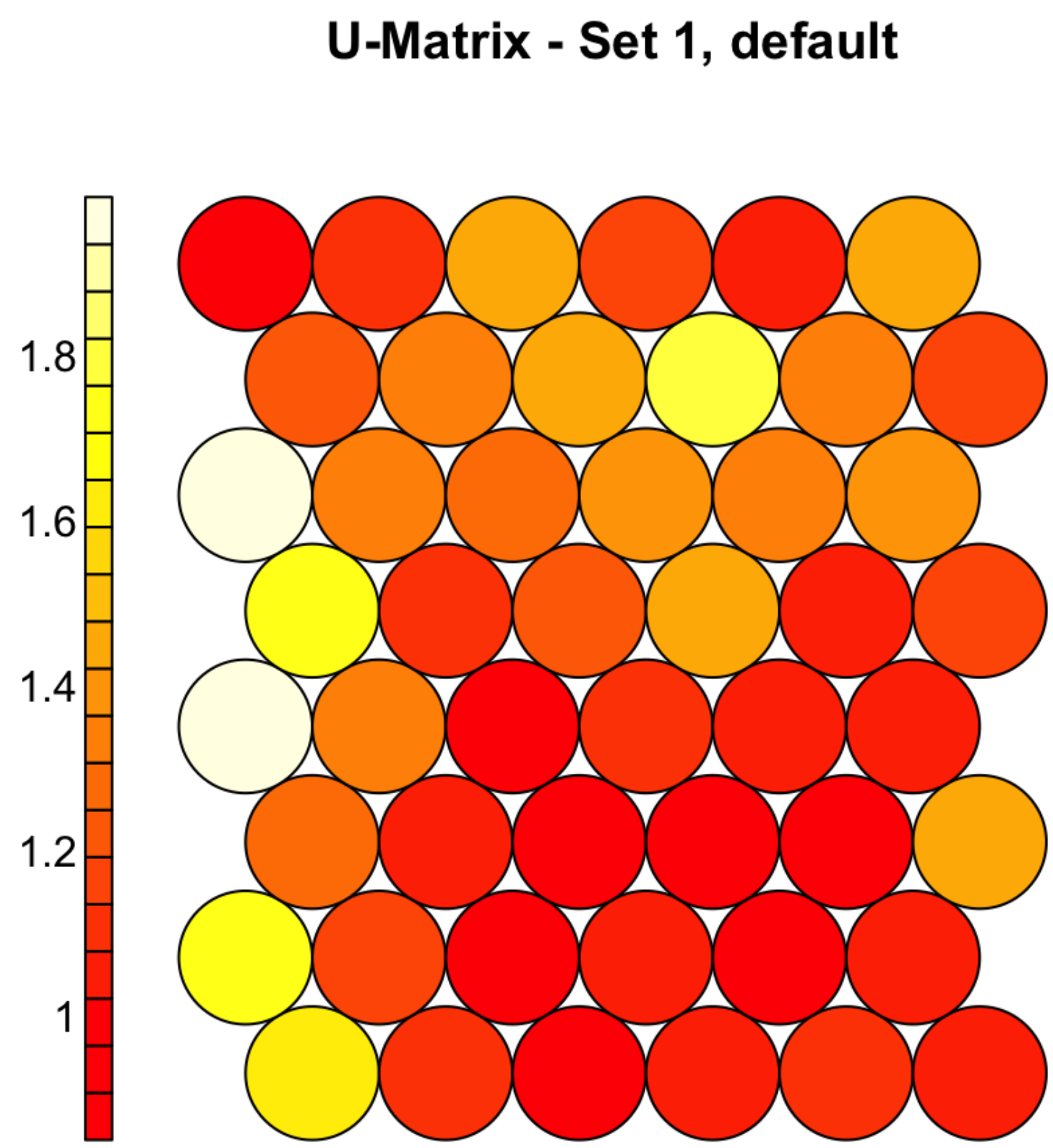


```
# 3.2. Assess Number of Iterations
```

```
#
________________________________________________________________________
____
# First, visualise training progress for the baseline model

plot(
  som_set1$changes, type = "l",
  xlab = "Iteration",
  ylab = "Mean distance change",
  main = "SOM training progress - Set 1, default")
```

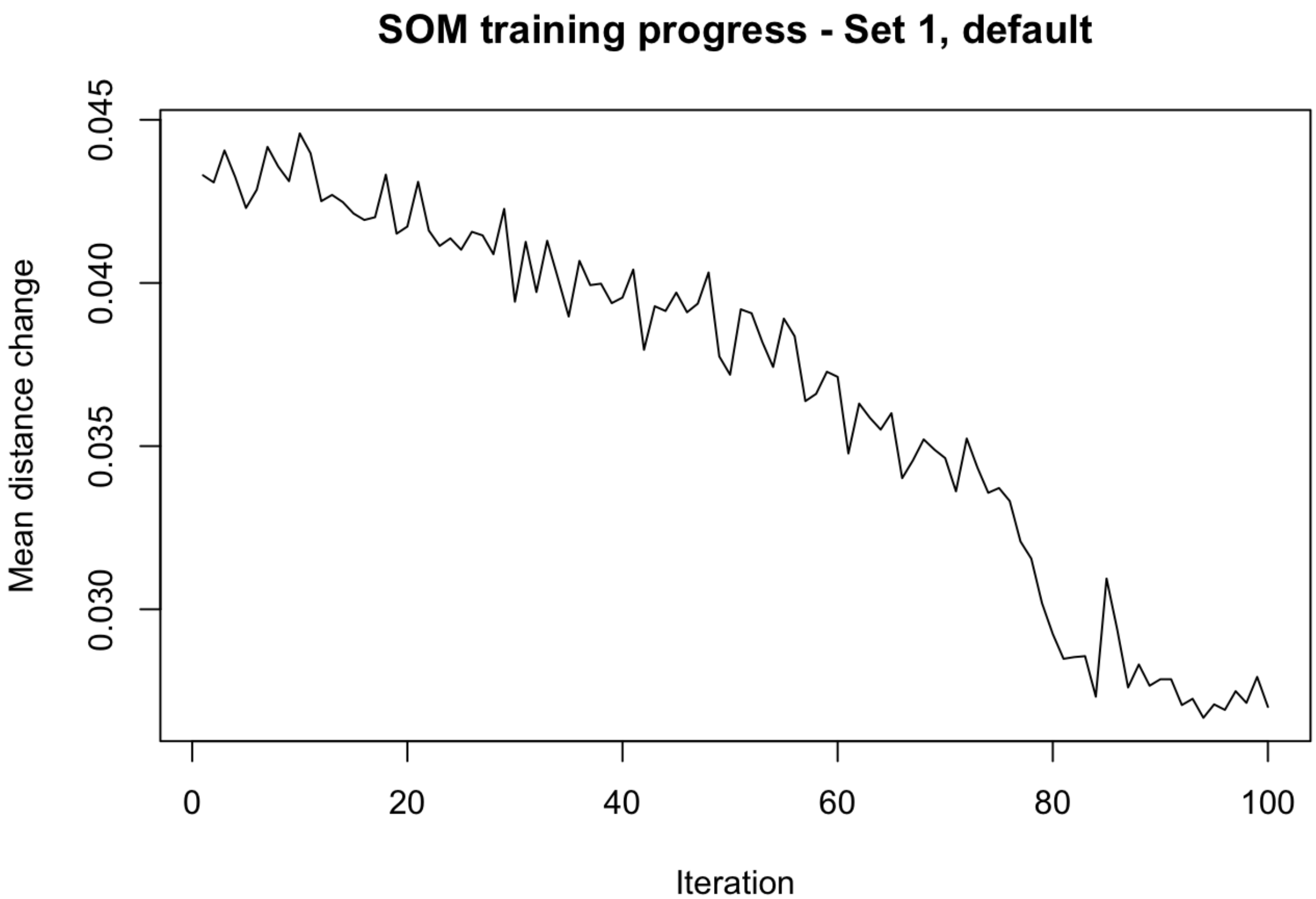


```
# Note: Evaluating the change in weights over iteration, we can see
that the map has not converged. Therefore, we will test a range of
iteration values to find the optimal balance
```

```
# 3.2.1. Test Multiple Iteration Values
# ————————————————————————————————————————————————————————————————
——

rlen_values <- c(100, 250, 500, 750, 1000)

som_results  <- vector("list", length(rlen_values))
quality_list <- vector("list", length(rlen_values))
changes_list <- vector("list", length(rlen_values))

# loop over iterations
for (i in seq_along(rlen_values)) {

  set.seed(201); som_tmp <- som(
    data_som,
    grid   = som_grid,
```

```
    rlen   = rlen_values[i])

  # collect quality metrics
  quality <- somQuality(som_tmp, data_som)
  quality_list[[i]] <- data.frame(
    rlen = rlen_values[i],
    QuantisationError = quality$err.quant,
    ExplainedVariance = quality$err.varratio,
    TopographicError  = quality$err.topo,
    KaskiLagusError   = quality$err.kaski
  )

  # collect training progress
  changes_list[[i]] <- data.frame(
    rlen      = rlen_values[i],
    iteration = seq_along(som_tmp$changes),
    change    = som_tmp$changes
  )

  som_results[[i]] <- som_tmp
}

# Combine results
results_df <- bind_rows(quality_list)
changes_df <- bind_rows(changes_list)

print(results_df)
```

```
##   rlen QuantisationError ExplainedVariance TopographicError
KaskiLagusError
## 1  100          1.575954             72.43        0.1627907
2.641053
## 2  250          1.479107             74.13        0.1295681
2.563495
## 3  500          1.487867             73.97        0.1262458
2.537048
## 4  750          1.449910             74.64        0.1627907
2.678227
## 5 1000          1.423530             75.10        0.1395349
2.625965
```

```
# 3.2.2. Visualise Training Progress
```

```
#
```

```
# Plot all training curves overlaid for comparison.

ggplot(changes_df, aes(x = iteration, y = change, colour = factor(rlen))) +
  geom_line(linewidth = 0.9) +
  labs(
    x = "Iteration",
    y = "Mean codebook update",
    title = "SOM training progress across iteration values",
    colour = "rlen"
  ) +
  theme_minimal()
```

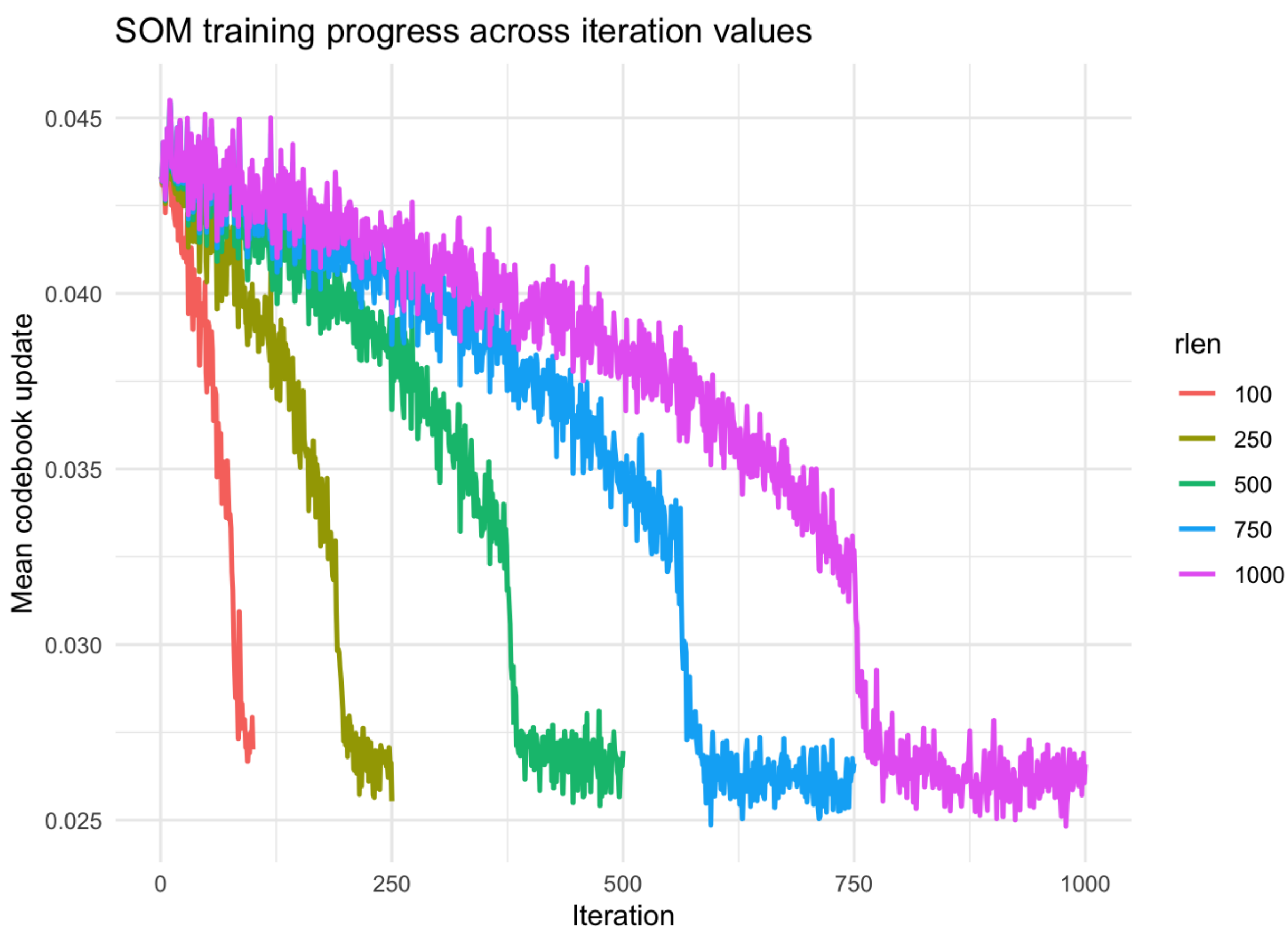


```
# 3.2.3. Select Optimal Iteration Count
```

```
# ____________________________________________________________________________

# Strategy: Choose the lowest number of iterations that achieves convergence (curve flattens)

optimal_rlen <- 750  # Based on visual inspection
```

```
# 3.2.4. Retrain with Optimised Iterations and Compare
```

```
# ————————————————————————————————————————————————————————————————
# After changing any SOM parameter (iterations, learning rate, radius, etc.), always re-inspect the grid visually. Visual inspection ensures the map remains interpretable and topographically sound.

set.seed(201); som_set2 <- som(
  data_som,
  grid = som_grid,
  alpha = c(0.05, 0.01), # default learning rate
  rlen = optimal_rlen) # default number of iterations

# Compare Hit Maps: Set 1 vs. Set 2
# ————————————————————————————————————————————————————————————————
par(mfrow = c(1, 2), mar = c(3, 3, 3, 1))

plot(som_set1, type = "counts", main = "Hit map - Set 1, default", palette.name = heat.colors) # Plot hit map

plot(som_set2, type = "counts", main = paste("Hit Map - Set 2 (rlen =", optimal_rlen,")"), palette.name = heat.colors) # Plot hit map
```

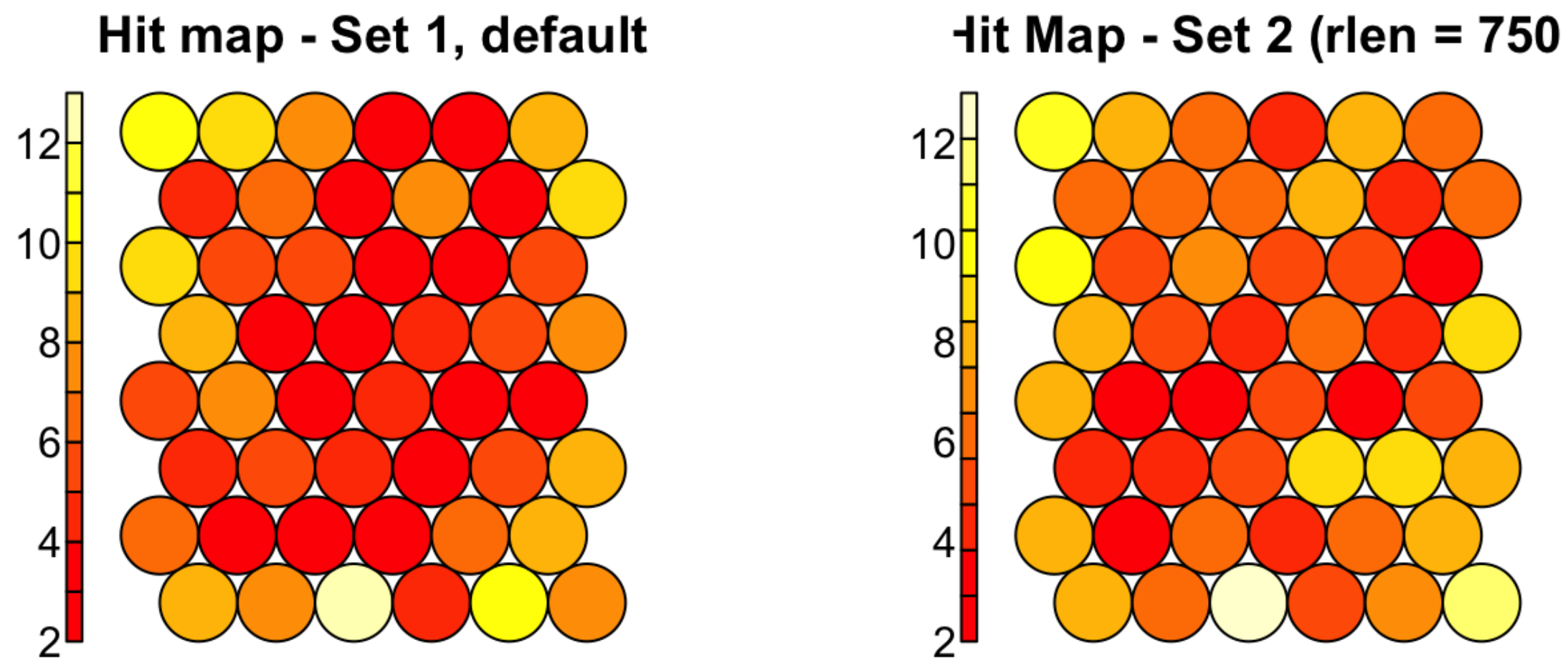


```
par(mfrow = c(1, 1))
```

```
# Compare U-Matrices: Set 1 vs. Set 2
#
par(mfrow = c(1, 2), mar = c(3, 3, 3, 1))

plot(som_set1, type = "dist.neighbours", main = "U-Matrix - Set 1, default", palette.name = heat.colors) # Plot U-Matrix

plot(som_set2, type = "dist.neighbours", main = paste("U-Matrix - Set 2 (rlen =", optimal_rlen,")"), palette.name = heat.colors) # Plot U-Matrix
```

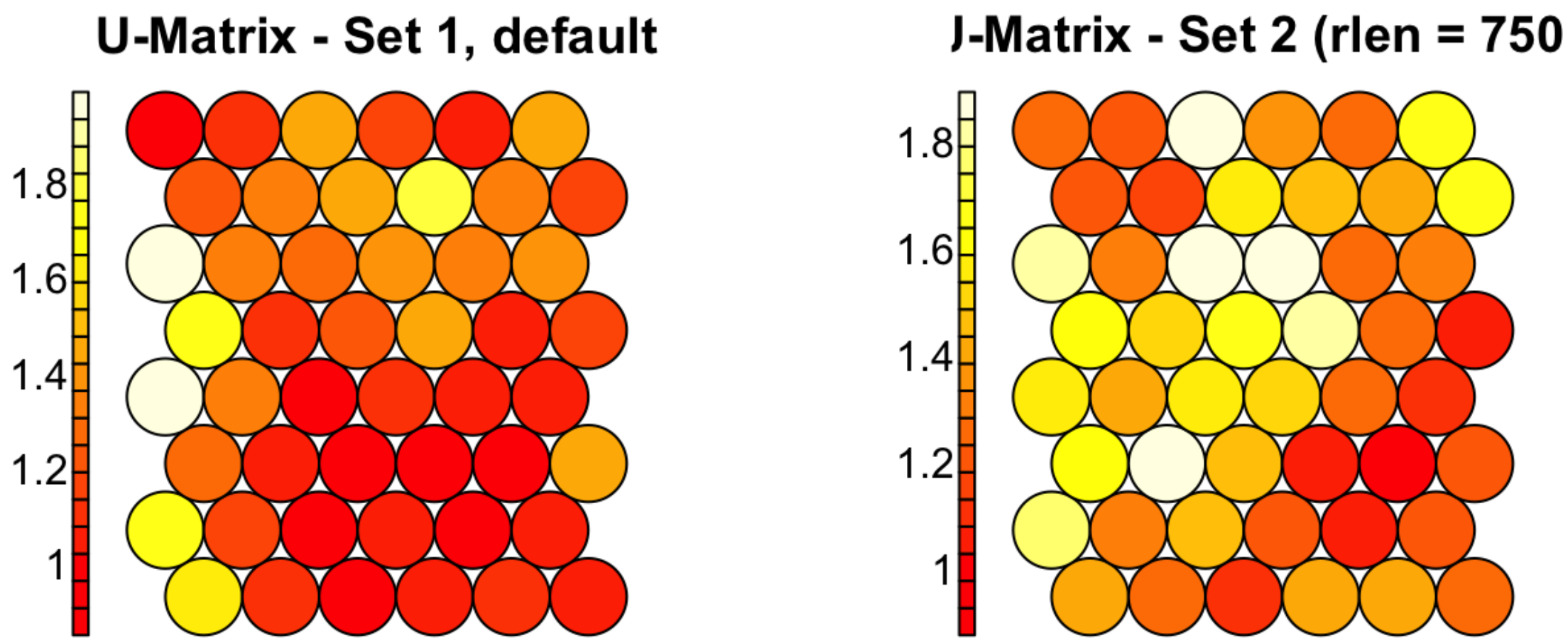


```
par(mfrow = c(1, 1))
```

```
# VISUAL ASSESSMENT: Set 2 (optimised) shows,
# - More even sample distribution across nodes (Hit Map),
# - Sharper, more defined cluster boundaries (U-Matrix)",
# - Better convergence and topographic structure
```

```
# 3.3. Optimise learning-rate (alpha)
```

```
#
___________________________________________________________________________________
____

rlen_fixed <- optimal_rlen # Use the optimised iteration count from Step 3.2

# Define grid of alpha values to test. Note that these ranges are tentative and should be
adjusted based on your specific dataset.

alpha1_vals <- seq(0.02, 0.1, by = 0.01) # Initial learning rate
alpha2_vals <- seq(0.005, 0.015, by = 0.005) # Final learning rate

alpha_grid <- expand.grid(
```

```
  alpha1 = alpha1_vals,
  alpha2 = alpha2_vals)

alpha_schedules <- split(alpha_grid, seq(nrow(alpha_grid)))
alpha_schedules <- lapply(alpha_schedules, function(row) c(row$alpha1, row$alpha2))

# 3.3.1. Train and Evaluate Models
# ────────────────────────────────────────────────────────────────────────────
────
results <- vector("list", length(alpha_schedules))

for (i in seq_along(alpha_schedules)) {
  a <- alpha_schedules[[i]]
  cat("Training SOM model", i, "of", length(alpha_schedules),
      "with alpha schedule:", paste(a, collapse = " -> "),
      ", rlen =", rlen_fixed, "\n")

  set.seed(201); som_tmp <- som(
    data_som,
    grid  = som_grid,
    rlen  = rlen_fixed,
    alpha = a)

# Evaluate quality
q <- somQuality(som_tmp, data_som)

results[[i]] <- data.frame(
  NumAlphaSteps     = length(a),
  Alpha1            = a[1],
  Alpha2            = a[2],
  AlphaSchedule     = paste(a, collapse = " -> "),
  Rlen              = rlen_fixed,
  QuantisationError = q$err.quant,
  ExplainedVariance = q$err.varratio,
  TopographicError  = q$err.topo,
  KaskiLagusError   = q$err.kaski,
  stringsAsFactors  = FALSE)
}
```

```
## Training SOM model 1 of 27 with alpha schedule: 0.02 -> 0.005 , rlen
= 750
```

```
## Training SOM model 2 of 27 with alpha schedule: 0.03 -> 0.005 , rlen
= 750
```

```
## Training SOM model 3 of 27 with alpha schedule: 0.04 -> 0.005 , rlen
= 750
## Training SOM model 4 of 27 with alpha schedule: 0.05 -> 0.005 , rlen
= 750
## Training SOM model 5 of 27 with alpha schedule: 0.06 -> 0.005 , rlen
= 750
## Training SOM model 6 of 27 with alpha schedule: 0.07 -> 0.005 , rlen
= 750
## Training SOM model 7 of 27 with alpha schedule: 0.08 -> 0.005 , rlen
= 750
## Training SOM model 8 of 27 with alpha schedule: 0.09 -> 0.005 , rlen
= 750
## Training SOM model 9 of 27 with alpha schedule: 0.1 -> 0.005 , rlen
= 750
## Training SOM model 10 of 27 with alpha schedule: 0.02 -> 0.01 , rlen
= 750
## Training SOM model 11 of 27 with alpha schedule: 0.03 -> 0.01 , rlen
= 750
## Training SOM model 12 of 27 with alpha schedule: 0.04 -> 0.01 , rlen
= 750
## Training SOM model 13 of 27 with alpha schedule: 0.05 -> 0.01 , rlen
= 750
## Training SOM model 14 of 27 with alpha schedule: 0.06 -> 0.01 , rlen
= 750
## Training SOM model 15 of 27 with alpha schedule: 0.07 -> 0.01 , rlen
= 750
## Training SOM model 16 of 27 with alpha schedule: 0.08 -> 0.01 , rlen
= 750
## Training SOM model 17 of 27 with alpha schedule: 0.09 -> 0.01 , rlen
= 750
## Training SOM model 18 of 27 with alpha schedule: 0.1 -> 0.01 , rlen
= 750
## Training SOM model 19 of 27 with alpha schedule: 0.02 -> 0.015 ,
rlen = 750
## Training SOM model 20 of 27 with alpha schedule: 0.03 -> 0.015 ,
rlen = 750
## Training SOM model 21 of 27 with alpha schedule: 0.04 -> 0.015 ,
rlen = 750
## Training SOM model 22 of 27 with alpha schedule: 0.05 -> 0.015 ,
rlen = 750
## Training SOM model 23 of 27 with alpha schedule: 0.06 -> 0.015 ,
rlen = 750
## Training SOM model 24 of 27 with alpha schedule: 0.07 -> 0.015 ,
rlen = 750
## Training SOM model 25 of 27 with alpha schedule: 0.08 -> 0.015 ,
rlen = 750
```

```
## Training SOM model 26 of 27 with alpha schedule: 0.09 -> 0.015 ,
rlen = 750
## Training SOM model 27 of 27 with alpha schedule: 0.1 -> 0.015 , rlen
= 750
```

```
tuning_results <- dplyr::bind_rows(results)
```

```
# Identify Default and Best Models
default_alpha_str <- "0.05 -> 0.01"
best_kl_idx <- which.min(tuning_results$KaskiLagusError)

tuning_results <- tuning_results %>%
  mutate(
    IsDefault = AlphaSchedule == default_alpha_str,
    IsBestKL  = row_number() == best_kl_idx)

# Sort table: Default first, then by best Kaski-Lagus error
tuning_results_sorted <- tuning_results %>%
  arrange(desc(IsDefault), KaskiLagusError)

# 3.3.2. Interactive Results Table
# ──────────────────────────────────────────────────────────────────────────
───
# We use 'reactable' to create a color-coded and interactive table for easy interpretation.

# This allows you to:
# - Sort by any column (click column header)
# - Identify best/worst performers at a glance via color intensity

make_color_pal <- function(colors) {
  function(x) rgb(colorRamp(colors)(x), maxColorValue = 255)
}

normalize <- function(x) {
  rng <- max(x) - min(x)
  if (rng == 0) return(rep(0.5, length(x)))
  (x - min(x)) / rng
}

alpha_table <- reactable(
  tuning_results_sorted %>% select(-IsDefault, -IsBestKL),
  sortable = TRUE,
  defaultPageSize = 10,
  columns = list(
```

```
    QuantisationError = colDef(
      style = function(value) {
        norm_val <- normalize(tuning_results_sorted$QuantisationError)
        idx <- match(value, tuning_results_sorted$QuantisationError)
        list(background = make_color_pal(c("white", "lightblue"))(norm_val[idx]))
      },
      format = colFormat(digits = 4)
    ),
    ExplainedVariance = colDef(
      style = function(value) {
        norm_val <- normalize(tuning_results_sorted$ExplainedVariance)
        idx <- match(value, tuning_results_sorted$ExplainedVariance)
        list(background = make_color_pal(c("white", "lightgreen"))(norm_val[idx]))
      },
      format = colFormat(digits = 4)
    ),
    TopographicError = colDef(
      style = function(value) {
        norm_val <- normalize(tuning_results_sorted$TopographicError)
        idx <- match(value, tuning_results_sorted$TopographicError)
        list(background = make_color_pal(c("white", "lightcoral"))(norm_val[idx]))
      },
      format = colFormat(digits = 4)
    ),
    KaskiLagusError = colDef(
      style = function(value) {
        norm_val <- normalize(tuning_results_sorted$KaskiLagusError)
        idx <- match(value, tuning_results_sorted$KaskiLagusError)
        list(background = make_color_pal(c("white", "lightyellow"))(norm_val[idx]))
      },
      format = colFormat(digits = 4)
    )
  ),
  rowStyle = function(index) {
    if (tuning_results_sorted$IsDefault[index]) {
      list(background = "#E6F3FF", fontWeight = "bold") # Blue for Default
    } else if (tuning_results_sorted$IsBestKL[index]) {
      list(background = "#E6FFE6", fontWeight = "bold") # Green for Best
    }
  }
)

print(alpha_table)

# ROW COLOUR SCHEME:
# - Blue row background: Default alpha schedule (0.05 → 0.01) for reference
```

```
# - Green row background: Best Kaski-Lagus error (lowest combined error)
# COLUMN COLOUR SCHEME (GRADEINTS):
# - Quantisation Error (Blue): Lower = better (lighter = better performance)
# - Explained Variance (Green): Higher = better (lighter = lower variance captured)
# - Topographic Error (Red): Lower = better (lighter = better map topology)
# - Kaski-Lagus Error (Yellow): Lower = better (lighter = better performance)

# 3.3.3. Select Best Alpha
# ——————————————————————————————————————————————————————————————————————————
optimal_alpha <- c(
  tuning_results$Alpha1[best_kl_idx],
  tuning_results$Alpha2[best_kl_idx])

# 3.3.4. Retrain with Optimised alpha and Compare to previous set
# ——————————————————————————————————————————————————————————————————————————
set.seed(201); som_set3 <- som(
  data_som,
  grid = som_grid,
  alpha = optimal_alpha, # default learning rate
  rlen = optimal_rlen) # default number of iterations

# Compare Hit Maps: Set 2 vs. Set 3
# ——————————————————————————————————————————————————————————————————————————
par(mfrow = c(1, 2), mar = c(3, 3, 3, 1))

plot(som_set2, type = "counts", main = paste("Hit Map - Set 2 (rlen =", optimal_rlen,")"),
palette.name = heat.colors) # Plot hit map

plot(som_set3, type = "counts", main = paste("Hit Map - Set 3\n(Alpha",
paste(optimal_alpha, collapse="->"), ")"), palette.name = heat.colors) # Plot hit map
```

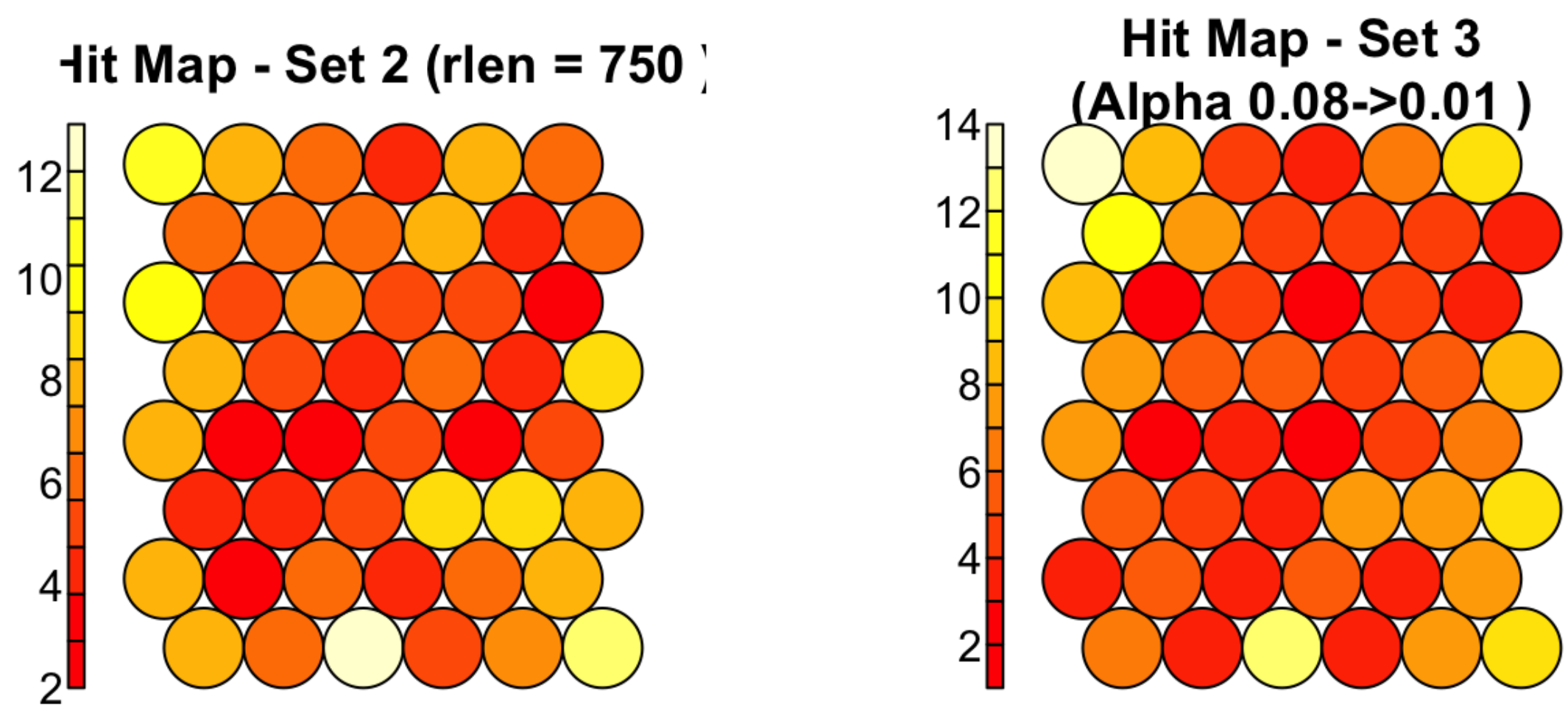


```
par(mfrow = c(1, 1))
```

```
# Compare U-Matrices: Set 2 vs. Set 3
# ------------------------------------------------------------------------

par(mfrow = c(1, 2), mar = c(3, 3, 3, 1))

plot(som_set2, type = "dist.neighbours", main = paste("U-Matrix - Set 2 (rlen =",
optimal_rlen,")"), palette.name = heat.colors) # Plot U-Matrix

plot(som_set3, type = "dist.neighbours", main = paste("Hit Map - Set 3\n(Alpha",
paste(optimal_alpha, collapse="->"), ")"), palette.name = heat.colors) # Plot U-Matrix
```

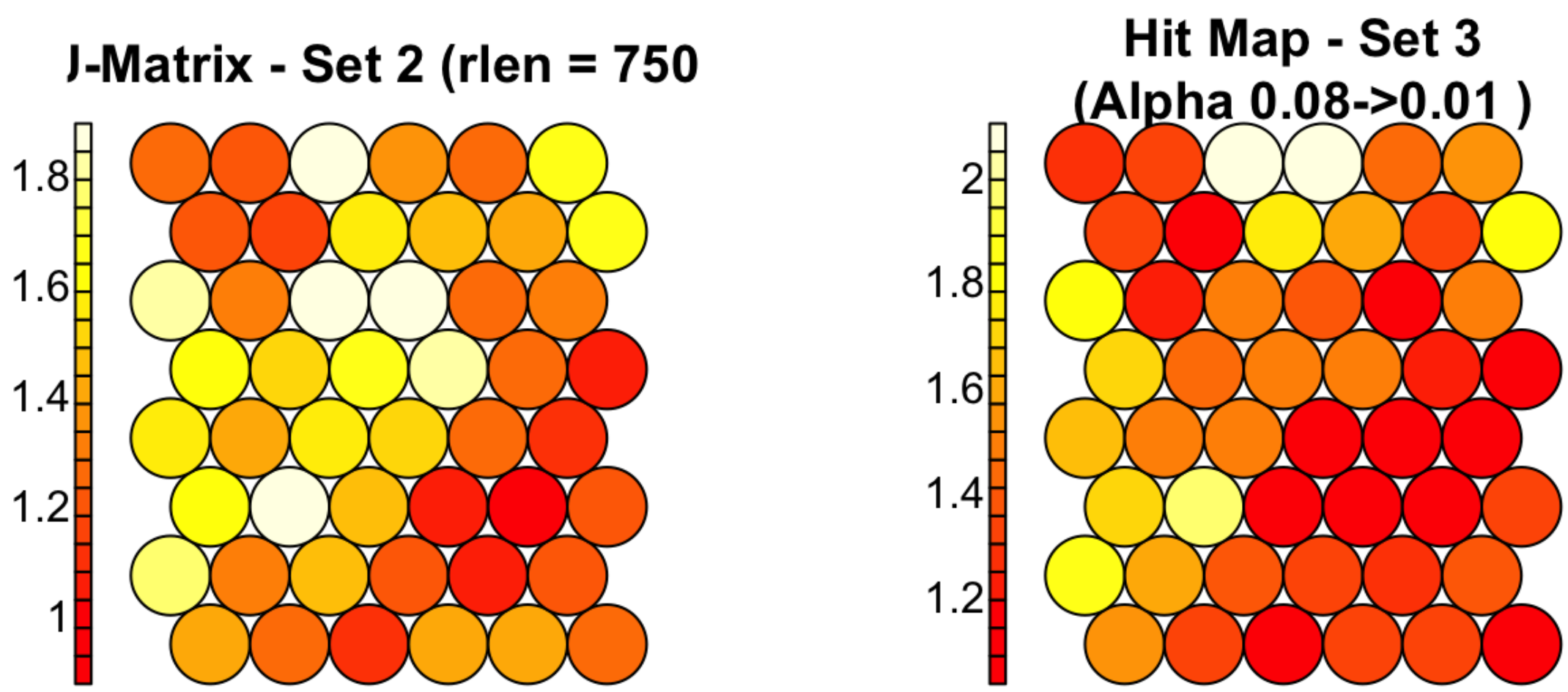


```
par(mfrow = c(1, 1))

# 3.4. Optimise Neighbourhood Radius
```

```
#
————————————————————————————————————————
alpha_fixed <- optimal_alpha
rlen_fixed  <- optimal_rlen

default_r <- som_set3$radius[1]

# Define radius range to test
sigma_range <- seq(floor(default_r - 2), ceiling(default_r + 2), by = 0.5)
if (!any(abs(sigma_range - default_r) < .001)) {
  sigma_vals <- sort(unique(c(sigma_range, default_r)))
} else {
  sigma_vals <- sigma_range
}

sigma_vals <- sigma_vals[sigma_vals > 0] # Ensure radius is positive
```

```
# 3.4.1. Train and Evaluate Models
#
────────────────────────────────────────────────────────────────
────
results <- vector("list", length(sigma_vals))

for (i in seq_along(sigma_vals)) {
  r <- sigma_vals[i]

  cat("Training SOM model", i, "of", length(sigma_vals),
      "with radius =", r,
      ", alpha =", paste(alpha_fixed, collapse = " -> "),
      ", rlen =", rlen_fixed, "\n")

  set.seed(201)
  som_tmp <- som(
    data_som,
    grid   = som_grid,
    rlen   = rlen_fixed,
    alpha  = alpha_fixed,
    radius = r)

# Evaluate quality
q <- somQuality(som_tmp, data_som)

results[[i]] <- data.frame(
  Radius            = r,
  Rlen              = rlen_fixed,
  QuantisationError = q$err.quant,
  ExplainedVariance = q$err.varratio,
  TopographicError  = q$err.topo,
  KaskiLagusError   = q$err.kaski)
}
```

```
## Training SOM model 1 of 9 with radius = 2 , alpha = 0.08 -> 0.01 ,
rlen = 750
```

```
## Training SOM model 2 of 9 with radius = 2.5 , alpha = 0.08 -> 0.01 ,
rlen = 750
## Training SOM model 3 of 9 with radius = 3 , alpha = 0.08 -> 0.01 ,
rlen = 750
## Training SOM model 4 of 9 with radius = 3.5 , alpha = 0.08 -> 0.01 ,
rlen = 750
## Training SOM model 5 of 9 with radius = 4 , alpha = 0.08 -> 0.01 ,
rlen = 750
```

```
## Training SOM model 6 of 9 with radius = 4.5 , alpha = 0.08 -> 0.01 ,
rlen = 750
## Training SOM model 7 of 9 with radius = 5 , alpha = 0.08 -> 0.01 ,
rlen = 750
## Training SOM model 8 of 9 with radius = 5.5 , alpha = 0.08 -> 0.01 ,
rlen = 750
## Training SOM model 9 of 9 with radius = 6 , alpha = 0.08 -> 0.01 ,
rlen = 750
```

```
tuning_results_radius <- bind_rows(results)
```

```
best_kl_idx_r <- which.min(tuning_results_radius$KaskiLagusError)

tuning_results_radius <- tuning_results_radius %>%
  mutate(
    IsDefault = (abs(Radius - default_r) < 0.01),
    IsBestKL  = (row_number() == best_kl_idx_r))

tuning_results_radius_sorted <- tuning_results_radius %>%
  arrange(desc(IsDefault), KaskiLagusError)

# 3.4.2. Interactive Results Table
# ——————————————————————————————————————————
radius_table <- reactable(
  tuning_results_radius_sorted %>% select(-IsDefault, -IsBestKL),
  sortable = TRUE,
  defaultPageSize = 10,
  columns = list(
    Radius = colDef(style = list(fontWeight = "bold")),
    QuantisationError = colDef(
      style = function(value) {
        norm_val <- normalize(tuning_results_radius_sorted$QuantisationError)
        idx <- match(value, tuning_results_radius_sorted$QuantisationError)
        list(background = make_color_pal(c("white", "lightblue"))(norm_val[idx]))
      }, format = colFormat(digits = 4)
    ),
    ExplainedVariance = colDef(
      style = function(value) {
        norm_val <- normalize(tuning_results_radius_sorted$ExplainedVariance)
        idx <- match(value, tuning_results_radius_sorted$ExplainedVariance)
        list(background = make_color_pal(c("white", "lightgreen"))(norm_val[idx]))
      }, format = colFormat(digits = 4)
    ),
```

```
    TopographicError = colDef(
      style = function(value) {
        norm_val <- normalize(tuning_results_radius_sorted$TopographicError)
        idx <- match(value, tuning_results_radius_sorted$TopographicError)
        list(background = make_color_pal(c("white", "lightcoral"))(norm_val[idx]))
      }, format = colFormat(digits = 4)
    ),
    KaskiLagusError = colDef(
      style = function(value) {
        norm_val <- normalize(tuning_results_radius_sorted$KaskiLagusError)
        idx <- match(value, tuning_results_radius_sorted$KaskiLagusError)
        list(background = make_color_pal(c("white", "lightyellow"))(norm_val[idx]))
      }, format = colFormat(digits = 4)
    )
  ),
  rowStyle = function(index) {
    if (tuning_results_radius_sorted$IsDefault[index]) {
      list(background = "#E6F3FF", fontWeight = "bold") # Blue for Default
    } else if (tuning_results_radius_sorted$IsBestKL[index]) {
      list(background = "#E6FFE6", fontWeight = "bold") # Green for Best
    }
  }
)

print(radius_table)

# 3.4.3. Select Best Radius
#
————————————————————————————————————————
——
optimal_radius <- tuning_results_radius$Radius[best_kl_idx_r]

# 3.4.4. Retrain with Optimised radius and Compare to previous set
#
————————————————————————————————————————
——
set.seed(201); som_set4 <- som(
  data_som,
  grid = som_grid,
  alpha = optimal_alpha,
  rlen = optimal_rlen,
  radius = optimal_radius)

# Compare Hit Maps: Set 3 vs. Set 4
```

```
#
————————————————————————————————————————————————————————————
———
par(mfrow = c(1, 2), mar = c(3, 3, 3, 1))

plot(som_set3, type = "counts", main = paste("Hit Map - Set 3\n(Default Radius =",
round(default_r, 2), ")"), palette.name = heat.colors)

plot(som_set4, type = "counts", main = paste("Hit Map - Set 4\n(Optimized Radius =",
round(optimal_radius, 2), ")"), palette.name = heat.colors)
```

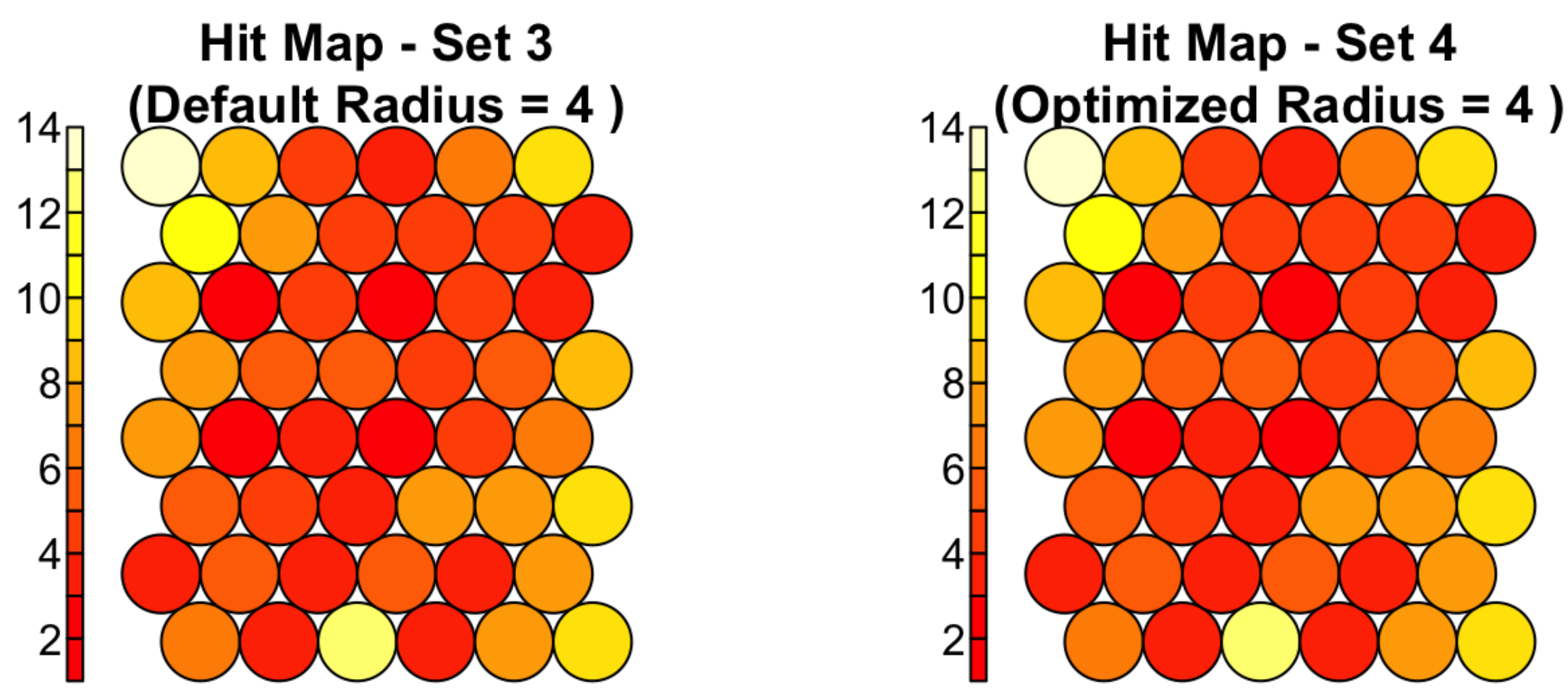


```
par(mfrow = c(1, 1))
```

```
# Compare U-Matrices: Set 3 vs. Set 4
#
————————————————————————————————————————————————————————————
———
par(mfrow = c(1, 2), mar = c(3, 3, 3, 1))

plot(som_set3, type = "dist.neighbours",
  main = "U-Matrix - Set 3",
```

```
  palette.name = heat.colors)

plot(som_set4, type = "dist.neighbours",
  main = "U-Matrix - Set 4",
  palette.name = heat.colors)
```

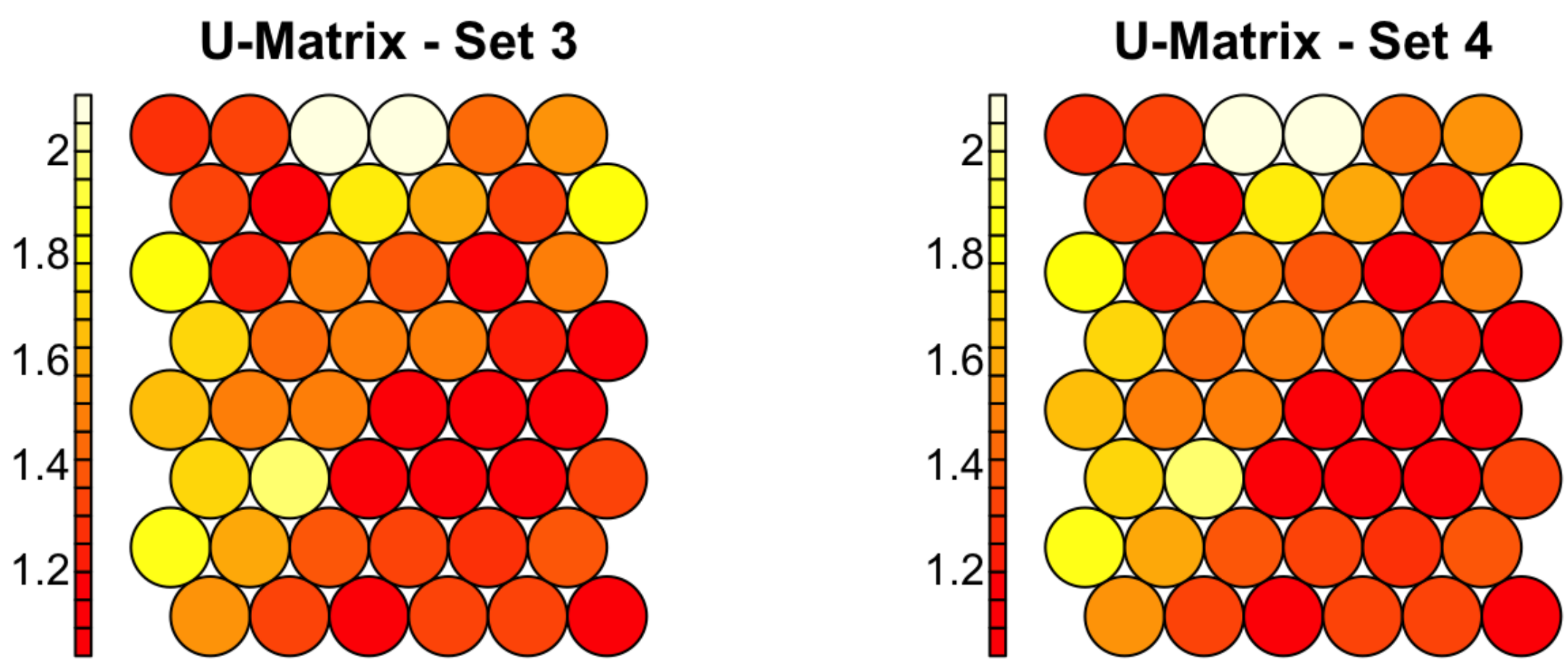


```
par(mfrow = c(1, 1))

# 3.5. Final Comparison of SOM Configurations
```

```
#
________________________________________________________________________
# We now compare three key models to demonstrate the impact of optimisation:
# Set 1: default parameters (baseline)
# Set 2: Default parameters + Optimised iterations
# Set 3: Optimised iterations, learning rate, and radius

# IMPORTANT!!!
# While Kaski-Lagus error is a useful combined metric, optimising based solely on numerical measures may not always yield the most interpretable or stable map.Always inspect the optimisation process visually:
```

```
# - Examine U-Matrix structure and cluster boundaries.
# - Check Hit Maps for balanced sample distribution.
# - Review training progress curves for smooth convergence.
# - Compare interpretability across configurations.
# Choose the simplest configuration that balances good metrics with clear, meaningful
structure. Sometimes a slightly higher error yields a more robust and interpretable map.

# 3.5.1. Build Summary Table
# ──────────────────────────────────────────────────────────────────────────
────
get_vec <- function(s, fields = c("alpha", "radius", "radii")) {
  for (f in fields) {
    v <- s[[f]]
    if (!is.null(v) && length(v) > 0) return(as.numeric(v))
  }
  numeric(0)
}

get_start_end <- function(v) {
  if (length(v) == 0) return(c(NA_real_, NA_real_))
  c(v[1], v[length(v)])
}

# Collect objects and labels
som_list  <- list(som_set1, som_set2, som_set3, som_set4)
set_names <- c("Set 1: Default",
               "Set 2: Optimised iterations",
               "Set 3: Optimised iterations and learning rate",
               "Set 4: Optimised iterations, learning rate, and radius")

# extract parameters
alpha_vals  <- t(vapply(som_list, function(s) get_start_end(get_vec(s, "alpha")),  c(0, 0)))
radius_vals <- t(vapply(som_list, function(s) get_start_end(get_vec(s, c("radius", "radii"))),
c(0, 0)))

# quality metrics
quality_list <- lapply(som_list, function(m) somQuality(m, data_som))

quality_summary <- data.frame(
  Set              = set_names,
  Rlen             = vapply(som_list, function(m) length(m$changes), numeric(1)),
  AlphaStart       = alpha_vals[, 1],
  AlphaEnd         = alpha_vals[, 2],
  RadiusStart      = radius_vals[, 1],
  RadiusEnd        = radius_vals[, 2],
```

```
  QuantisationError = vapply(quality_list, `[[`, numeric(1), "err.quant"),
  ExplainedVariance = vapply(quality_list, `[[`, numeric(1), "err.varratio"),
  TopographicError  = vapply(quality_list, `[[`, numeric(1), "err.topo"),
  KaskiLagusError   = vapply(quality_list, `[[`, numeric(1), "err.kaski"),
  check.names = FALSE)

quality_summary |>
  dplyr::mutate(dplyr::across(where(is.numeric), ~ round(.x, 4))) |> print(row.names =
FALSE)
```

```
##                                                        Set Rlen
AlphaStart
```

```
##                                             Set 1: Default  100
0.05
##                              Set 2: Optimised iterations  750
0.05
##              Set 3: Optimised iterations and learning rate  750
0.08
##  Set 4: Optimised iterations, learning rate, and radius  750
0.08
##  AlphaEnd RadiusStart RadiusEnd QuantisationError ExplainedVariance
##      0.01           4         0            1.5760             72.43
##      0.01           4         0            1.4499             74.64
##      0.01           4         0            1.4361             74.88
##      0.01           4         0            1.4361             74.88
##  TopographicError KaskiLagusError
##            0.1628          2.6411
##            0.1628          2.6782
##            0.1096          2.5148
##            0.1096          2.5148
```

```
# 3.5.2. Visual Comparison: U-Matrices
```

```
#
________________________________________________________________________
____

par(mfrow = c(1, 3), mar = c(2, 2, 3, 1))
plot(som_set1, type = "dist.neighbours", main = "Set 1: Default", palette.name = heat.colors)
plot(som_set2, type = "dist.neighbours", main = "Set 2: Default + iterations", palette.name =
heat.colors)
plot(som_set4, type = "dist.neighbours", main = "Set 4: Optimised parameters",
palette.name = heat.colors)
```

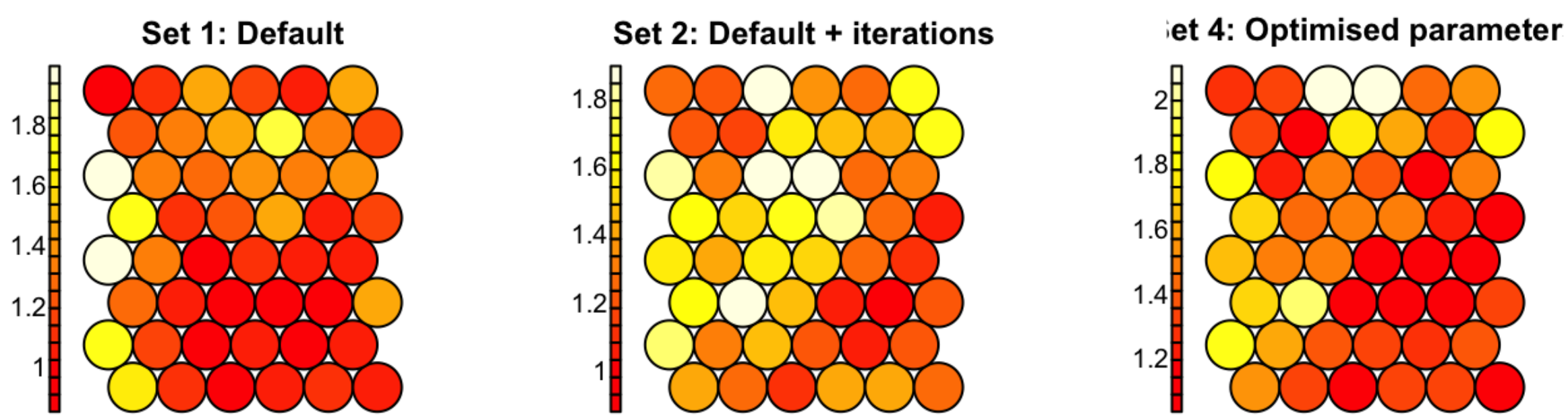


```
par(mfrow = c(1, 1))
```

```
# 3.5.3. Visual Comparison: Hit Maps with Counts
#
————————————————————————————————————————
——
plot_counts_with_labels <- function(som_obj, main = "Counts") {
  plot(
    som_obj, type = "counts",
    palette.name = function(n) rev(heat.colors(n)),
    main = main
  )
  # Add text labels for counts
  n_units <- nrow(som_obj$codes[[1]])
  counts  <- table(factor(som_obj$unit.classif, levels = seq_len(n_units)))
  counts  <- as.integer(counts)
  xy      <- som_obj$grid$pts

  # Scale text size by count (larger count = larger text)
  cex_scale <- if (max(counts) > 0) 0.8 + 0.6 * (counts / max(counts)) else 0.8
```

```
  labels    <- ifelse(counts > 0, counts, "")

  text(xy[, 1], xy[, 2], labels = labels, cex = cex_scale, col = "black", font = 2)
}

par(mfrow = c(1, 3), mar = c(2, 2, 3, 1))
plot_counts_with_labels(som_set1, "Set 1: Default")
plot_counts_with_labels(som_set2, "Set 2: Default + iterations")
plot_counts_with_labels(som_set4, "Set 4: Optimised parameters")
```

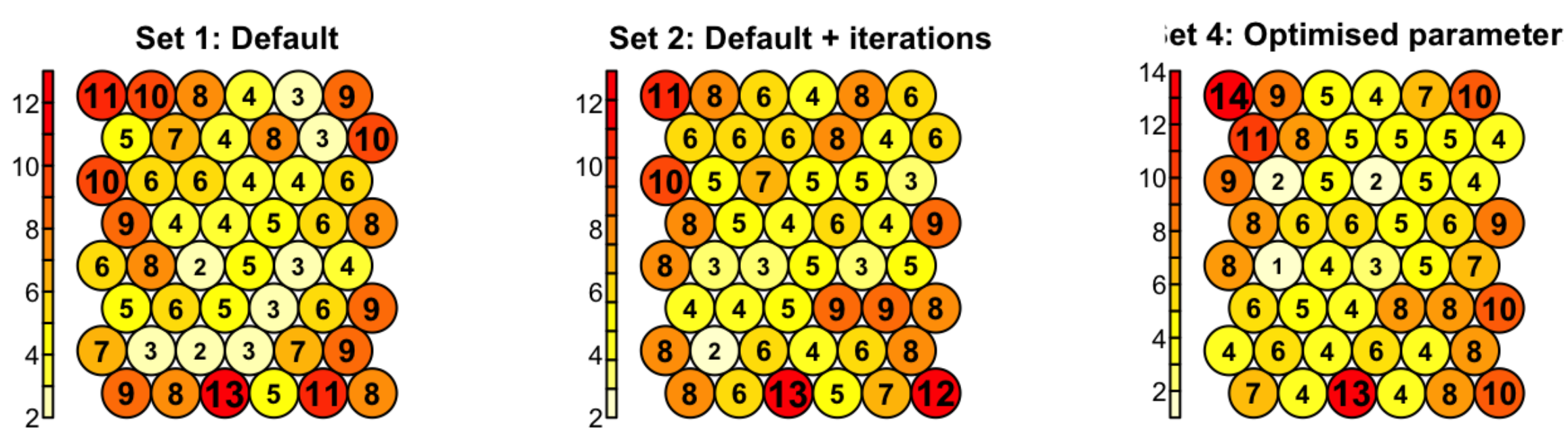


```
par(mfrow = c(1, 1))
```

```
# CONCLUSION
# The optimised model (Set 4) should demonstrate:
# 1. Lower Quantisation Error (better fit to data).
# 2. Lower Topographic Error (better preservation of data structure).
# 3. Clearer U-Matrix boundaries (more distinct clusters).
# 4. More balanced sample distribution in the Hit Map.
```

```
# ==============================================================
```

```
#  ------  4. Interpreting the SOM: CLUSTERING THE SOM  -----
# ==============================================================

# In this tutorial, we focus on clustering approach as one way of further analysing and interpreting the SOM.

# WHAT ARE WE CLUSTERING?
# Each SOM node represents a profile shared by groups of individuals that has been learned from the data. We want to group these similar nodes together, to identify larger patterns or profiles that occur frequently among participants.

# 4.1. Determine Optimal Number of Clusters (k)
# ______________________________________________________________________________
# To do this, we first need to decide how many groups to make. We use k-means clustering, and test a range of possible group counts (k).

nodes <- som_set4$codes[[1]]  # Each row is a SOM node's profile (not a participant)
wss_function <- function(k, data) {
  kmeans(data, centers = k, nstart = 25)$tot.withinss
}
k_values <- 2:15
wss_values <- sapply(k_values, function(k) wss_function(k, nodes))

# Plot to find a sensible number of clusters
plot(k_values, wss_values,
     type = "b",
     pch = 19,
     frame = FALSE,
     xlab = "Number of clusters K",
     ylab = "Total within-cluster sum of squares",
     main = "Elbow Method for Optimal K"); grid()
```

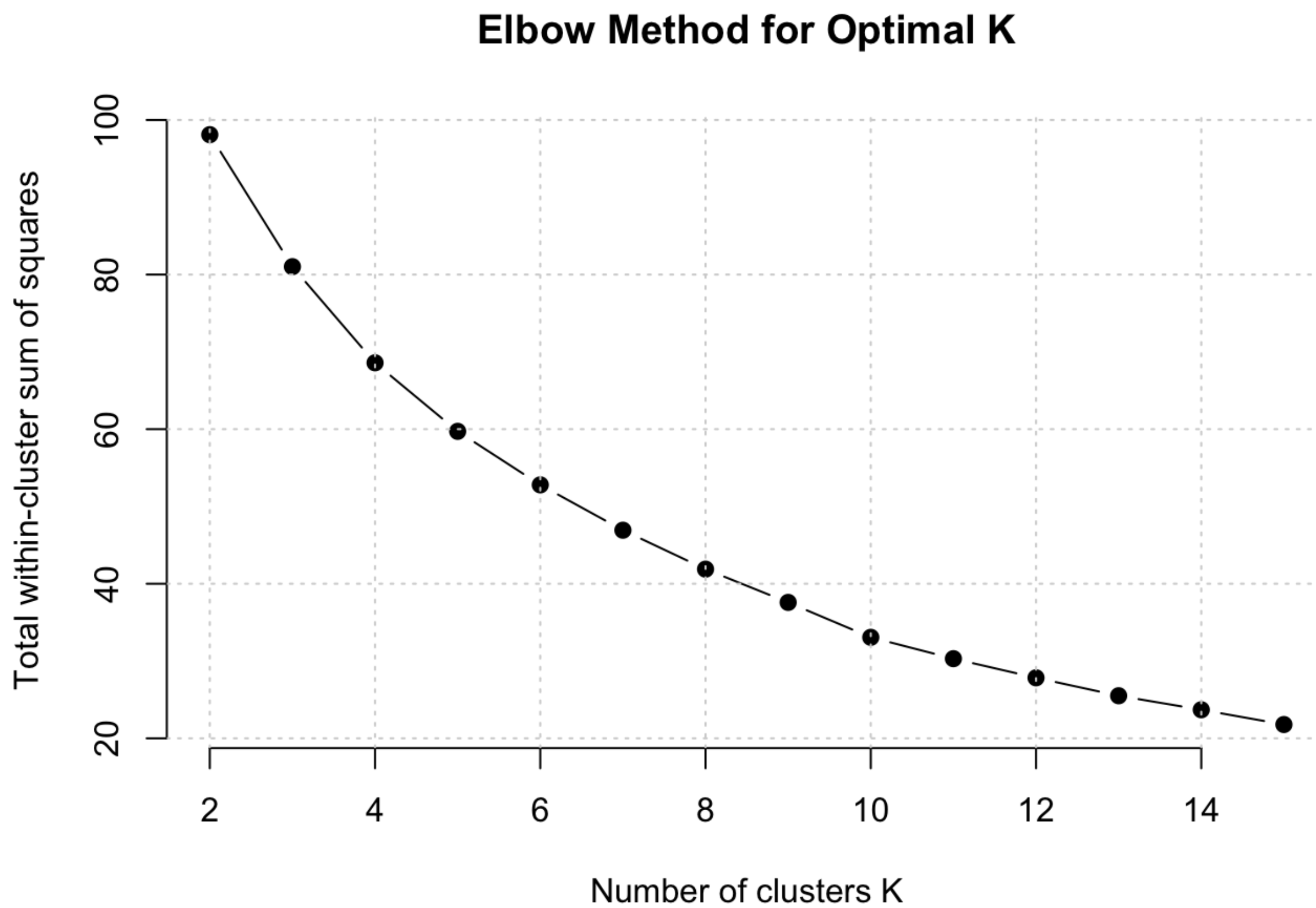


```
# One way to assess the optimal number of clusters is by examining this plot. # The x axis (k) shows how many groups you're dividing the SOM nodes into. The y axis shows the within-cluster Sum of Squares (WCSS),measuring how tight the nodes are from their centre within the clusters. Lower WCSS means that nodes in each group are more similar to each other (representing better grouping).
```

As k increases, WCSS always decreases. This is because more groups equals smaller groups, with less distances between participants. BUT: At some point, adding more groups gives only tiny improvements. This is where you see the curve flatten out.

Look for the 'elbow' point, where adding more groups does not reduce the spread within each group much. This gives a balance between too few and too many groups. In this plot, the curve shows a steeper drop from k=2 to k=4, then flattens considerably after k=4, suggesting the primary elbow is around k=4. However, the drop from k=2 to k=3 is substantial. Therefore, choosing either k=3 or k=4 would be reasonable. We chose k=3 as a more parsimonious solution that captures the main structure.

k <- 3 # Number of clusters decided on

```
# 4.2. Cluster SOM Nodes
# ————————————————————————————————————————————————————————————
——
set.seed(201); km_result <- kmeans(nodes, centers = k, nstart = 25)
node_clusters <- km_result$cluster

# 4.3. Assign Each Participant to a Group
# ————————————————————————————————————————————————————————————
——
# Each participant is already connected to one SOM node (their BMU). We then assign
these nodes into larger clusters and to assign each participant to a cluster, we look at which
cluster their match nodes belongs to

participant_nodes <- som_set4$unit.classif # Which node each participant matched to
participant_clusters <- node_clusters[participant_nodes] # Which cluster that node is in

# Attach group labels to your raw data for further analysis
data_with_groups <- as.data.frame(data_som)
data_with_groups$SOM_Group <- participant_clusters

# 4.4. Summarise the Clusters Statistics
# ————————————————————————————————————————————————————————————
——
group_summary <- data_with_groups %>%
  group_by(SOM_Group) %>%
  summarise(
    n = n(),
    across(where(is.numeric), list(
      mean = ~ mean(.x, na.rm=TRUE),
      sem  = ~ sd(.x, na.rm=TRUE) / sqrt(sum(!is.na(.x)))
    ), .names = "{.col}_{.fn}")
  )

print(group_summary)
```

```
## # A tibble: 3 × 18
```

```
##   SOM_Group     n Anhedonia_mean Anhedonia_sem Low.energy_mean
Low.energy_sem
##       <int> <int>          <dbl>         <dbl>           <dbl>
<dbl>
## 1         1    64           1.09        0.0794            1.58
0.109
```

```
## 2         2    86           2.07        0.0807            2.59
0.0649
## 3         3   151           1.32        0.0654            2.12
0.0563
## # ℹ 12 more variables: Hopelessness_mean <dbl>, Hopelessness_sem
<dbl>,
## #   Dysphoria_mean <dbl>, Dysphoria_sem <dbl>, Loss.of.interest_mean
<dbl>,
## #   Loss.of.interest_sem <dbl>, Low.self.worth_mean <dbl>,
## #   Low.self.worth_sem <dbl>, Meaninglessness_mean <dbl>,
## #   Meaninglessness_sem <dbl>, n_mean <dbl>, n_sem <dbl>
```

```
# 4.5. Silhouette Scores
```

```
#
———————————————————————————————————————
——
cluster_cols <- c("#E69F00", "#56B4E9", "#009E73", "#F0E442")[1:k]
# We calculate the average silhouette width across all nodes. Higher values indicate better-
defined, more separated clusters.

sil_scores <- silhouette(node_clusters, dist(nodes))
avg_silhouette <- mean(sil_scores[, "sil_width"])

plot(sil_scores,
    main = paste("Silhouette Plot for k =", k),
    col = cluster_cols,
    border = NA, do.col.sort = TRUE)
```

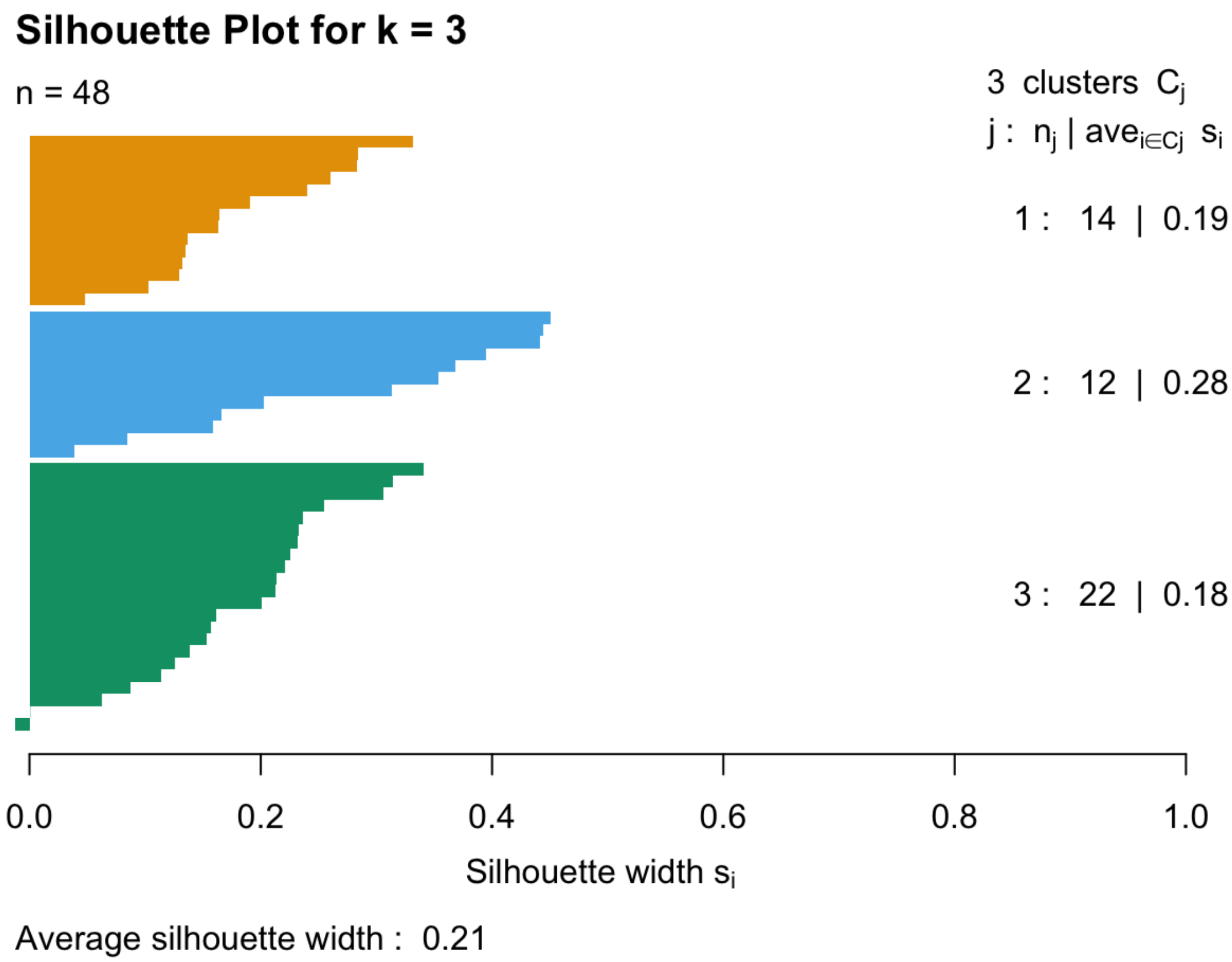


```
# In the plot, each horizontal bar represents one SOM node. Bars are
grouped by colour (one colour per cluster). Bars extending far to the
right represents nodes that fit well in their clusters. Bars that are
short or extend to the left represent nodes that fit poorly.
```

On the right side of the plot, you'll see the average silhouette score for each cluster and the number of nodes in each cluster. The overall average silhouette score is shown at the bottom.

# Print summary statistics
cat("\n=== SILHOUETTE ANALYSIS ===\n")

```
##
## === SILHOUETTE ANALYSIS ===
```

```
cat("Overall Average Silhouette Width:", round(avg_silhouette, 4),
"\n\n")
```

```
## Overall Average Silhouette Width: 0.2083
```

```
# 4.6. Using Prototypes to Interpret Clusters
```

```
#
________________________________________________________________________________
____

# When Silhouette scores are low, to better interpret the clusters we may want to find the node whose profile is most 'typical' for that cluster. This can be done by finding the node that the shortest distance to all other nodes in that cluster (also called medoid).

medoid_indices <- numeric(k)
medoid_profiles <- matrix(NA, nrow = k, ncol = ncol(nodes))

for (i in 1:k) {
  this_group_nodes <- which(node_clusters == i)
  node_profiles <- nodes[this_group_nodes, , drop = FALSE]
  group_center <- colMeans(node_profiles)
  dists <- apply(node_profiles, 1, function(row) sqrt(sum((row - group_center)^2)))
  medoid_idx <- which.min(dists)
  medoid_indices[i] <- this_group_nodes[medoid_idx]
  medoid_profiles[i, ] <- node_profiles[medoid_idx, ]
}

medoid_summary <- as.data.frame(medoid_profiles)
colnames(medoid_summary) <- colnames(nodes)
medoid_summary <- medoid_summary %>%
  mutate(SOM_Group = 1:k, Node = medoid_indices) %>%
  select(SOM_Group, Node, everything())

print(medoid_summary)
```

```
##   SOM_Group Node Anhedonia Low.energy Hopelessness Dysphoria
Loss.of.interest
## 1         1    8  1.067416   1.015858    1.7311728  2.205081
1.4992021
## 2         2   38  1.854535   2.295833    2.0893029  2.680570
2.2187100
## 3         3   11  1.707904   1.979872    0.5306458  1.738552
0.8733745
##   Low.self.worth Meaninglessness
## 1       1.656807       1.6863219
## 2       2.016489       2.4172206
## 3       1.246483       0.3034066
```

```
# 4.7. Visualise the Clusters on the Grid
```

```
#
________________________________________________________________________
____
cluster_cols <- c("#E69F00", "#56B4E9", "#009E73", "#F0E442")[1:k]
unit_background <- cluster_cols[node_clusters]

par(mar = c(2, 2, 3, 1))
plot(
  som_set4,
  type  = "mapping",
  bgcol = unit_background,
  pch   = NA,
  main  = paste("SOM Groups (k =", k, ")")
)
add.cluster.boundaries(som_set4, node_clusters, lwd = 3, col = "black")

# Show medoid nodes with a star
medoid_coords <- som_set4$grid$pts[medoid_indices, ]
points(medoid_coords, pch = 8, cex = 2, col = "white", lwd = 2)
points(medoid_coords, pch = 8, cex = 2, col = "black", lwd = 1)

legend(
  "bottom",
  legend = c(paste("Group", 1:k), "Representative node"),
  fill   = c(colors, NA),
  pch    = c(rep(NA, k), 8),
  col    = c(rep(NA, k), "black"),
  bty    = "n", ncol = k + 1, cex = 0.8)
```

```
## Warning in rect(left, top, r, b, angle = angle, density = density,
...):
## supplied color is neither numeric nor character
## Warning in rect(left, top, r, b, angle = angle, density = density,
...):
## supplied color is neither numeric nor character
## Warning in rect(left, top, r, b, angle = angle, density = density,
...):
## supplied color is neither numeric nor character
## Warning in rect(left, top, r, b, angle = angle, density = density,
...):
## supplied color is neither numeric nor character
```

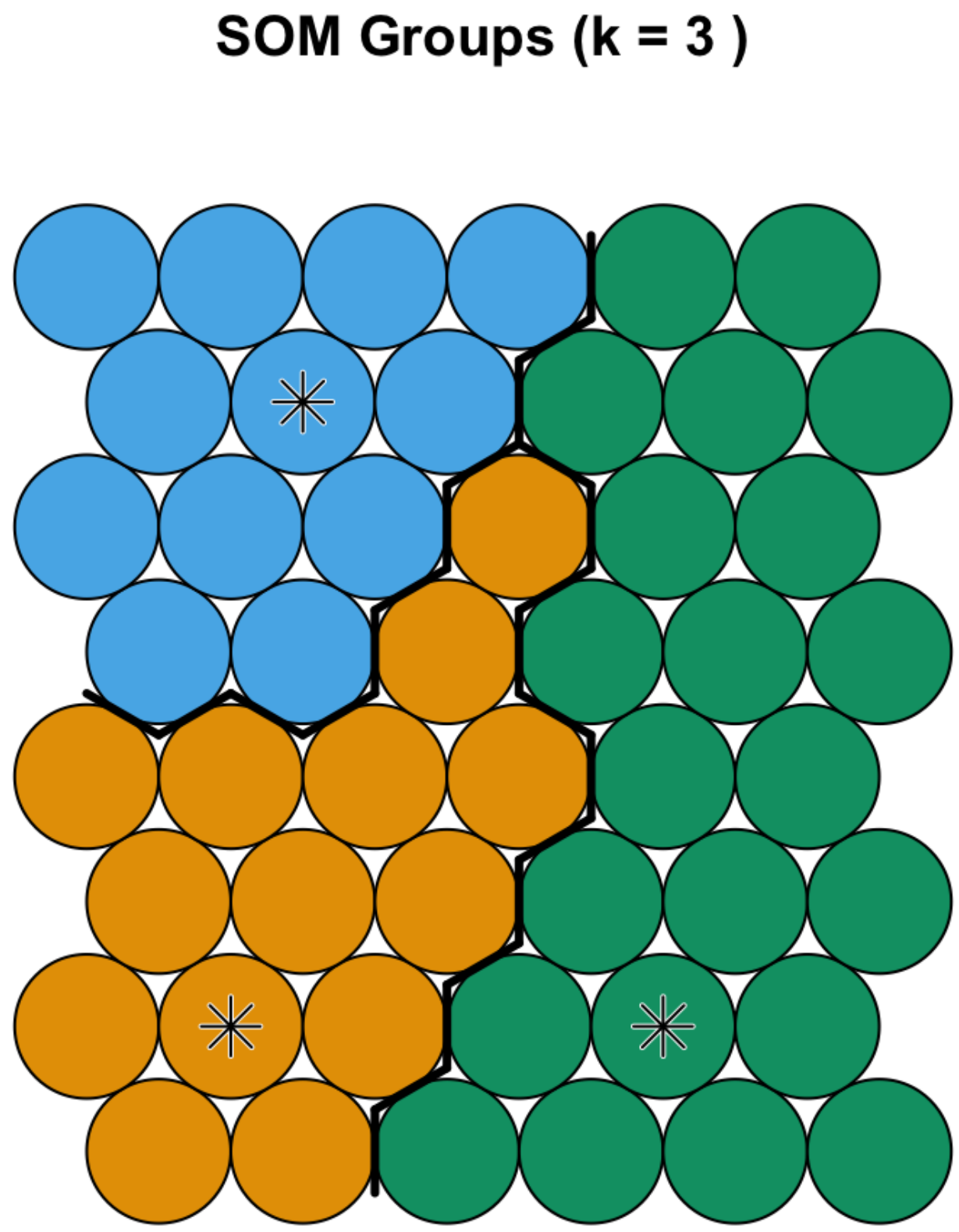


```
# 4.8. Visualise Cluster Profiles (Radar Charts)
```

```
#
________________________________________________________________________
____
# Radar plots can be used to visualise the symptom profile of each
# cluster's average or its representative node.

# 4.8.1. Prepare Data for Radar Plot
#
________________________________________________________________________
____
medoid_data <- medoid_summary %>%
  select(-SOM_Group, -Node)

# Define Max/Min anchors (Scale is 0-3)
max_min <- data.frame(
  matrix(c(rep(3, ncol(medoid_data)), rep(0, ncol(medoid_data))),
         nrow = 2, byrow = TRUE)
)
colnames(max_min) <- colnames(medoid_data)
```

```
radar_data <- bind_rows(max_min, medoid_data)

# 4.8.2. Plot Clusters
#
______________________________________________________________________________
____
cluster_cols <- c("#E69F00", "#56B4E9", "#009E73", "#F0E442")[1:k]

par(mfrow = c(1, k), mar = c(1, 1, 2, 1))

for (i in 1:k) {
  row_idx <- i + 2

  fmsb::radarchart(
    radar_data[c(1, 2, row_idx), ], # Select Max, Min, and current Cluster
    axistype = 1,
    seg = 3,
    caxislabels = 0:3,
    pcol = cluster_cols[i],
    pfcol = grDevices::adjustcolor(cluster_cols[i], alpha.f = 0.3),
    plwd = 2,
    cglcol = "grey",
    cglty = 1,
    axislabcol = "grey",
    vlcex = 0.8,
    title = paste("Cluster", i, "Profile")
  )
}
```

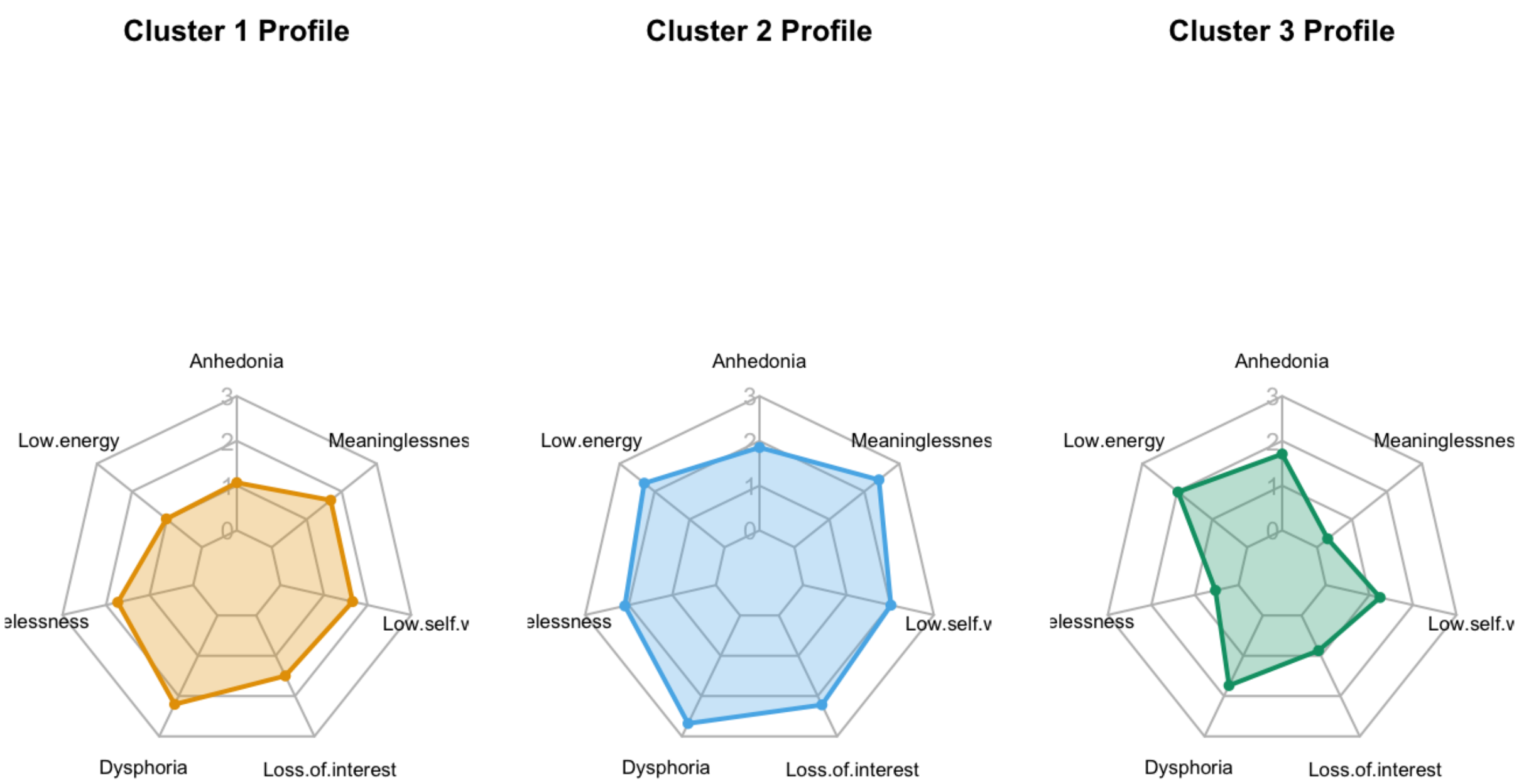


```
par(mfrow = c(1, 1))
```

Supplementary Materials

Checking Reliability and Validating the SOM

Assessing the Reliability of the SOM

Similar to other machine-learning approaches, training a SOM involves a degree of inherent non-linear variability, and thus, results can vary slightly each time the model is run. Therefore, it is important to assess its reliability to ensure that the patterns identified by the SOM are meaningful and not simply due to chance. One way to do this, similar to a method called hold-out validation, involves training the SOM on a randomly selected subset of the data rather than the full dataset (commonly around 70%, depending on the dataset size and number of variables). The same analysis is then performed on this subset, and the process is repeated multiple times, each time using a different random subset. By comparing the results across these subsets, we can evaluate whether the SOM produces consistent patterns. If the main structures remain similar across all runs, the SOM can be considered reliable, capturing real patterns in the data rather than artefacts of random variation.

Validating k-means clustering on SOM

We propose testing the stability of SOM clusters using a bootstrap approach, a common method used in clustering validation (e.g., Binhimd & Kalantan, 2023; Yu et al., 2019). The idea is that we repeatedly draw random subsamples from the main dataset (e.g., hundreds of times), retrain the SOM model on each subsample, and examine how consistently individuals are assigned to the same clusters. For each cluster, stability is quantified using the Jaccard Index, calculated as the proportion of participants from each original cluster who were reassigned to the same cluster (Hennig, 2008). This index reflects how reliably a cluster is reproduced across resampled datasets, with values below 0.5 indicating poor stability, while values above 0.75 indicate good stability.

For Review Only

It is important to note that during SOM retraining, it is possible that cluster assignment changes as the topology is variable and cluster naming is arbitrary – a phenotype could be named Cluster A and occupy the top left of the map in one iteration, and in another iteration, occupy the bottom right while being named Cluster 3, all while retaining the same participants. To account for arbitrary label switching, we suggest using the Hungarian method (Kuhn, 1955) to align clusters across iterations based on maximal participant overlap (example in Supplementary Materials code).

Supplementary References

Binhimd, S., & Kalantan, Z. (2023). Bootstrap approach for clustering method with applications. *International journal of advanced and applied sciences*, *10*(3), 189-195. https://doi.org/10.21833/ijaas.2023.03.023

Hennig, C. (2008). Dissolution point and isolation robustness: Robustness criteria for general cluster analysis methods. *Journal of Multivariate Analysis*, *99*(6), 1154-1176. https://doi.org/10.1016/j.jmva.2007.07.002

Kuhn, H. W. (1955). The Hungarian method for the assignment problem. *Naval Research Logistics Quarterly*, *2*(1-2), 83-97. https://doi.org/10.1002/nav.3800020109

Yu, H., Chapman, B., Di Florio, A., Eischen, E., Gotz, D., Jacob, M., & Blair, R. H. (2019). Bootstrapping estimates of stability for clusters, observations and model selection. *Computational Statistics*, *34*(1), 349-372. https://doi.org/10.1007/s00180-018-0830-y

For Review Only

Supplementary Text - Comparing Self-Organising Maps to Common Methods

Self-Organising Maps (SOMs) are introduced in the main text as one of several options for analysing psychological data. In this supplement, we apply four widely used alternatives (Exploratory Factor Analysis, network analysis, k-means clustering, and Latent Profile Analysis) to the same DASS-21 depression dataset used in the main text. Each method reflects a different analytic philosophy (variable- vs person-centred; dimensional vs categorical; linear vs non-parametric), and applying them side by side to identical data highlights what is gained, what is lost, and when each approach can be useful. Table 1 in the main text summarises the methods and how they differ from SOMs.

All analyses were conducted in R v4.4.1 (R Core Team, 2021). EFA used the *psych* v2.4.6.26 package (Revelle, 2026), with parallel analysis for factor retention, maximum likelihood extraction, and oblimin rotation. Network analysis used *bootnet* v1.6 (Epskamp et al., 2018) to estimate a regularised partial-correlation network via EBICglasso ($\gamma = 0.5$). PCA + *k*-means using the *stats* v4.4.1 package (R Core Team, 2021). LPA was estimated with *mclust* v6.1.2 (Scrucca et al., 2016), selecting the best model using BIC.

Exploratory Factor Analysis

Exploratory Factor Analysis (EFA) is one of the most commonly used methods in psychological research (Watkins, 2018). EFA examines how variables covary to identify common factors and determine how many underlying factors are needed to account for their shared variance. The result is a small set of latent dimensions, each defined by the items that load most strongly on that factor. In the present sample, parallel analysis suggested a two-factor solution (Figure S1). The first factor was anchored by *meaninglessness* and *hopelessness*, reflecting a cognitive-existential dimension. The second was anchored by *dysphoria*, *low energy*, and *anhedonia*, reflecting an affective-somatic dimension. *Low self-worth* and *loss of interest* did not load cleanly on either factor.

Figure S1

Exploratory Factor Analysis of the DASS-21 Depression Items

For Review Only

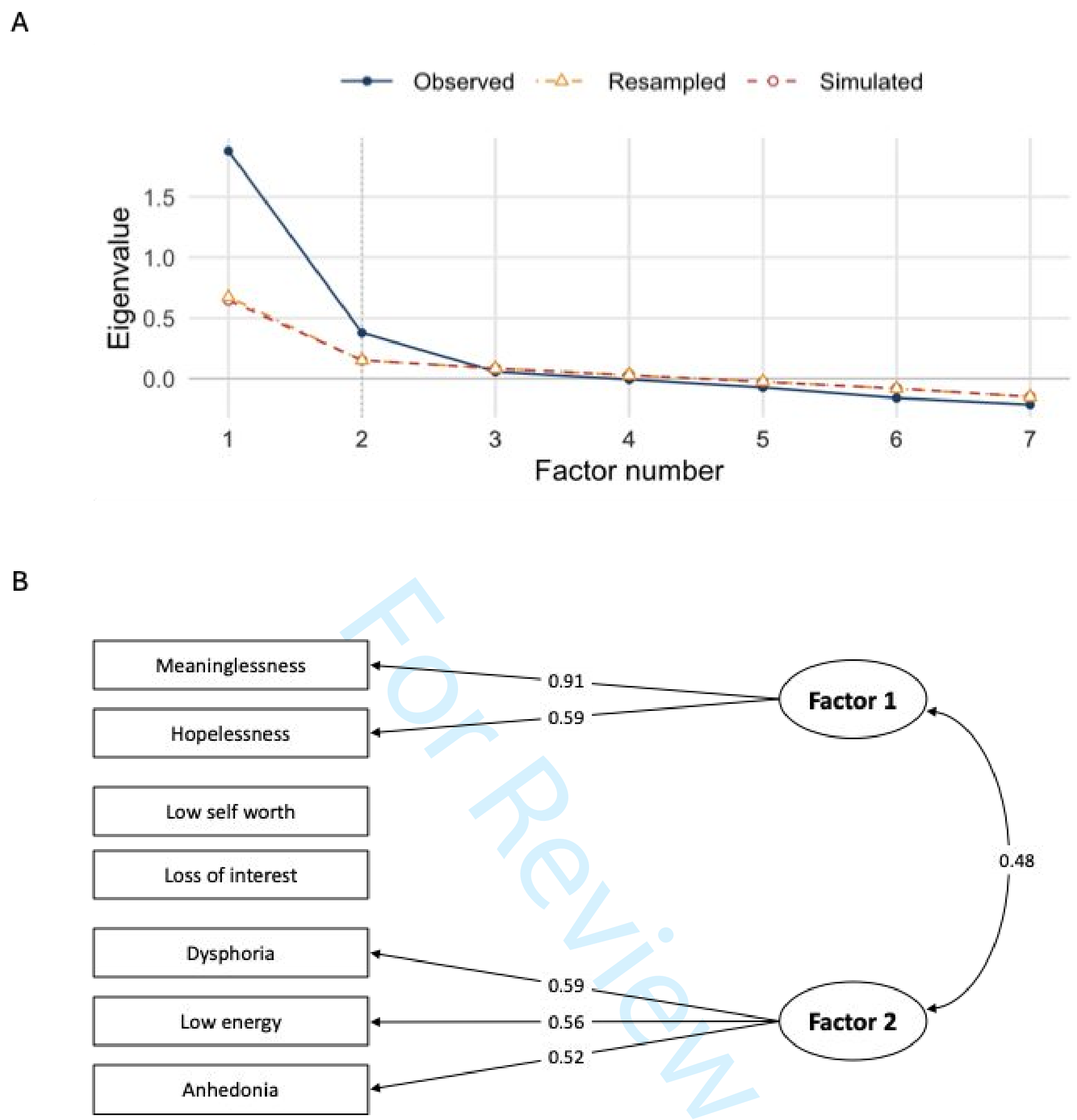

Note. Panel A shows a line plot used to decide how many factors to keep in an analysis. On the y-axis, the numbers 1 to 7 mark the possible factor numbers. The x-axis values show how much variance each factor explains (eigenvalues). A solid blue line shows the values from the real data. This line is highest at Factor 1 (above 2), lower at Factor 2 (around 1), and then close to zero from Factors 3 to 7. Two red dashed lines show the values that would be expected by chance from comparison datasets. For Factors 1 and 2, the blue line is clearly higher than both red lines, suggesting only the first two factors explain more variance than would be expected by chance, while additional factors do not add meaningful information. Panel B presents the factor structure of the depression items. Two latent factors (ML1 and ML2) are shown, each linked to subsets of items that load most strongly on it. The two latent factors are shown as moderately positively related, indicating that individuals who score higher on one factor also tend to score higher on the other, although the overlap is not complete.

For Review Only

This two-factor structure echoes a long-standing distinction in the depression literature between somatic-affective and cognitive symptoms (Beck, 1996; Krause et al., 2010), and suggests that the seven DASS-21 items capture two related but distinguishable aspects of depression. A practical implication is that a single sum score across the seven items may blend two content domains that, while related, carry partly separate information. Notably, the two factors are moderately correlated ($r = 0.48$), meaning that people who score high on one tend to score high on the other. This is consistent with the dimensional view of depression where cognitive and affective-somatic symptoms tend to rise and fall together, but not perfectly, leaving room for individual differences in which side is more prominent (Haslam et al., 2020). Additionally, the ambiguous loadings of *low self-worth* and *loss of interest* should not be interpreted as a lack of relevance of these items. Instead, it may indicate that these items do not fit neatly within either factor.

EFA is useful when the goal is to *identify how variables are grouped into underlying factors and how these factors relate to one another* (Watkins, 2018). For example, this can be used when developing or validating a measure or testing whether a construct is best represented by one or multiple dimensions (Flora & Flake, 2017). Consequently, it is one of the most established methods in psychometrics and provides a clear, interpretable description of latent structure at the variable level.

However, EFA assumes that a uniform underlying factor structure applies across all individuals in the sample (Howard & Hoffman, 2018). In other words, EFA is a variable-centred approach describing how variables relate at the population level, but nothing about how *people* differ in their symptom patterns. For example, a patient presenting with high hopelessness and preserved energy becomes difficult to distinguish under EFA. The method would summarise them as elevated on Factor 1 and low on Factor 2, collapsing their seven-item profile into the same two scores as anyone else with similar factor scores, even if their individual symptom patterns differ in clinically important ways. EFA is also a linear approach (Watkins, 2018) where each person's responses are summarised as weighted sums of factor scores, which means that symptoms which *interact*, where the clinical meaning depends on their combination rather than their separate elevations, cannot be represented. This is unlike SOMs, which are a person-centred and non-parametric method, retaining

For Review Only

the symptom configuration of each individual rather than reducing it to scores on universal dimensions (Kohonen, 2001).

Network analysis

A more granular understanding of how variables relate to one another can be obtained using network analysis (Borsboom & Cramer, 2013; Epskamp et al., 2018). Network analysis represents variables as nodes in a graph and their associations as edges. Rather than asking which subgroups of people exist, it asks which symptoms are most tightly linked to which others, after controlling for all other symptoms. In the present data, the analysis produced a dense network of symptoms that visualises their interconnections (Figure S2). The network revealed a strong link between hopelessness and meaninglessness. Dysphoria was found to be connected to several other symptoms, including moderately strong connections to low energy and anhedonia, suggesting it sits near the centre of the affective–somatic part of the network. Other symptoms (such as low self-worth and loss of interest) have mainly weaker, lighter connections, indicating less central roles in this estimated symptom structure. Clinically, this can be meaningful, suggesting that highly connected symptoms, such as meaninglessness and dysphoria, may be more likely, if successfully treated, to produce reductions in other symptoms via the network (Borsboom, 2017), although the causal interpretation of such centrality remains debated and should be treated cautiously when generalising to individual cases (Bringmann et al., 2019).

Figure S2

For Review Only

Network Analysis of the DASS-21 Depression Item

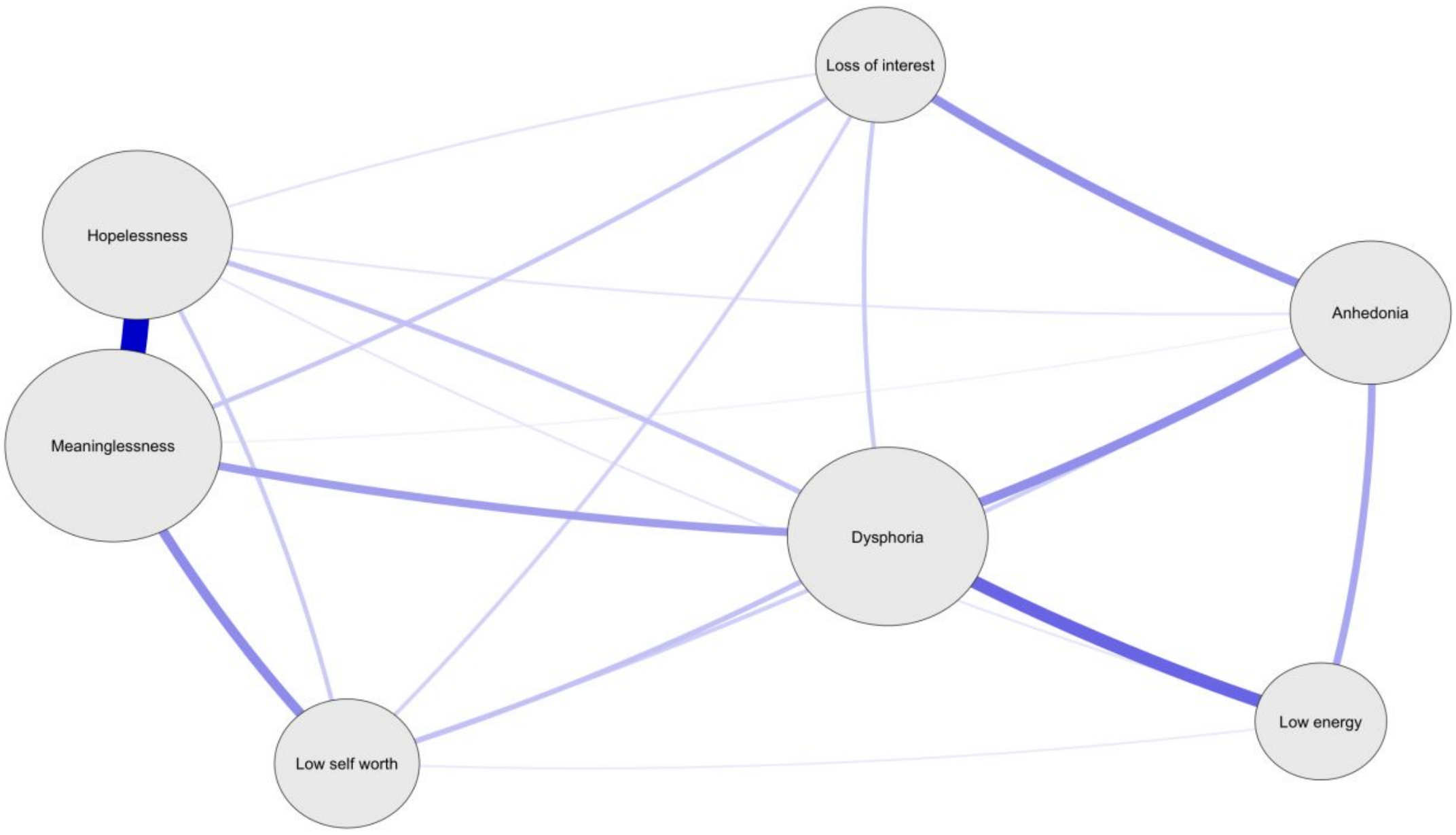

Note. Nodes represent depression symptoms, and edges represent estimated conditional associations between symptoms after controlling for all others. Thicker and darker blue edges indicate stronger associations. Node size is proportional to the sum of the absolute edge weights attached to it, with larger nodes indicating stronger connections to the rest of the network.

Review Only

Network analysis is well-suited to identify *how variables relate to one another*, and for the identification of "hub" variables (Epskamp et al., 2018). It is also a natural choice for transdiagnostic research, where symptom-level relationships may be more meaningful than diagnostic categories (Borsboom & Cramer, 2013). However, like EFA, cross-sectional network analysis as used here is variable-centred, showing how variables relate to one another at the sample level, but do not tell us how individuals differ (Fried & Cramer, 2017). More recent developments, such as time-series or individualised network models, hold promise for capturing individual dynamics, but their use for drawing robust conclusions about person-level differences remains constrained (Hoekstra et al., 2023). For example, it does not show how symptom profiles vary across patients, or which patient showed elevated levels of which symptoms. Our network analysis would tell a clinician that meaninglessness is centrally connected in the population-level architecture of depression, but it does not show which subgroups of patients present with a profile in which meaninglessness is elevated in

combination with particular other symptoms (for example, preserved energy, as found in the main text). SOM and network analysis are therefore complementary rather than competing, one a graph of variables, the other a map of people.

PCA + k-means

Moving toward a more person-centred approach, clustering methods are typically used for profile identification (Gao et al., 2023). One widely used strategy combines Principal Components Analysis (PCA) with *k*-means clustering. PCA first reduces the original items into a smaller number of principal components that capture as much of the total variance in the data as possible. Methods such as *k*-means then group participants into a prespecified number of clusters based on how similar their scores are on these principal components.

In the present data, two components were retained, which together explained 51.9% of the variance (Figure S3A). In other words, the seven symptoms of depression were compressed and summarised along two dimensions. Participants were then plotted in the two-dimensional space defined by these components and grouped into clusters using *k*-means. Guided by the scree plot and cluster-quality indices (Figure S3B), we selected a three-cluster solution. This yielded three groups of participants positioned according to their scores on the two principal components (Figure S3C).

Figure S3

Principal Components Analysis and k-means Clustering of the DASS-21 Depression Items

For Review Only

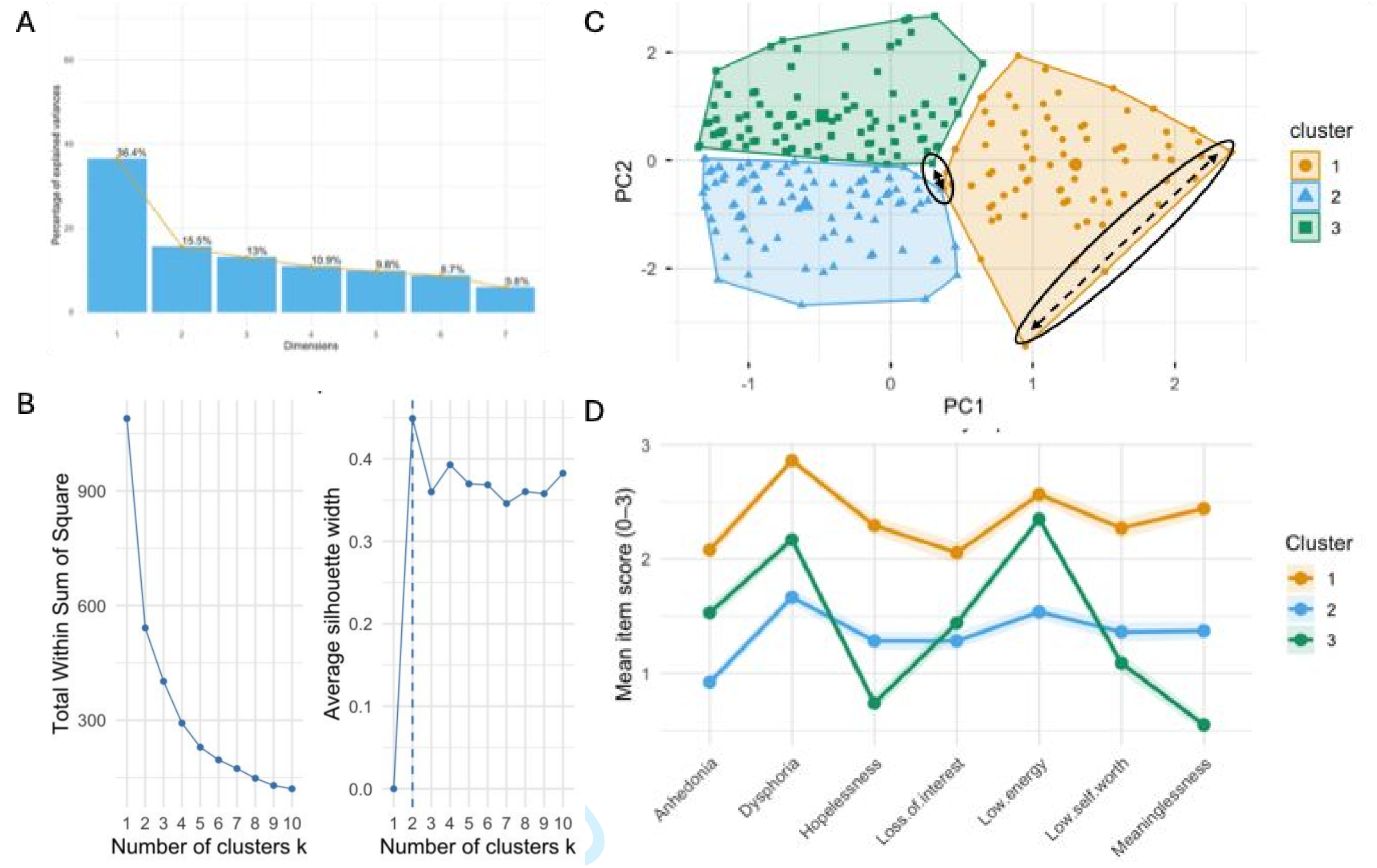

Note. The figure contains four panels that summarise a PCA plus k-means clustering analysis of seven depression symptoms. Panel A shows the proportion of variance explained by each principal component. The first two components account for most of the variance, with a marked drop after the second component, supporting a two-component solution. Panel B shows an elbow plot of within-cluster sum of squares (left) and the average silhouette width (right). The elbow suggests diminishing returns beyond three to four clusters, whereas the silhouette index is maximised for two clusters. Within this 2–4 cluster range, we selected a three-cluster solution to facilitate comparison with the three clusters identified by the SOM in the main text. Panel C plots participants in the space defined by the first two principal components (PC1 and PC2), with points coloured and shaped according to the three k-means clusters and cluster boundaries outlined. Panel D presents mean scores for each of the seven depression symptoms within each cluster.

Then, the average symptom levels in each cluster can be examined to characterise the resulting profiles (Figure S3D). On average, participants in the first cluster showed elevated scores

across all symptoms; participants in the second cluster showed relatively elevated dysphoria and milder symptoms; and participants in the third cluster showed moderate elevations dominated by anhedonia, dysphoria, and fatigue.

PCA is particularly useful when a dataset includes many variables, and the aim is to condense them into a smaller number of summary dimensions that still capture most of the differences between individuals (Jolliffe & Cadima, 2016). For example, it can reduce dozens of questionnaire items into a few components that summarise respondents' overall symptoms or personality profiles, or compress thousands of fMRI voxels into a small, workable set of dimensions. However, the same reduction that makes this method powerful is also a limitation, particularly in applied settings. The resulting principal components are linear combinations of items that, while mathematically neat, can be theoretically abstract (PC1 and PC2 in Figure S3C) and cannot be easily communicated or described in terms of well-defined constructs (Mazziotta & Pareto, 2019). Because PCA captures only linear combinations of variables, it may also fail to represent non-linear relationships between variables, potentially obscuring nuanced profiles that could be substantively meaningful (Jolliffe & Cadima, 2016).

Additionally, while k-means clustering may provide insights into profiles by averaging item scores within each cluster (see Figure S3D), it imposes categorical boundaries between clusters. As reiterated in the main text, this ignores the dimensional nature of psychological constructs and limits the ability to capture variability within groups and similarities at boundary regions (De Boeck et al., 2023; Haslam et al., 2020). In such cases, it may produce counterintuitive results, where, as illustrated in Figure S3C, two participants sitting close together on either side of a cluster boundary (full arrows) are assigned different profiles, while two participants positioned far apart within the same cluster (dashed arrow) are treated as belonging to the same group. SOMs solve these issues by maintaining the original variables and their interpretation, classifying participants based on the similarity of their full symptom profiles, and arranging them on a continuous two-dimensional space that preserves the topology of the data (Kohonen, 2001, 2013).

For Review Only

Latent Profile Analysis

An increasingly popular person-centred alternative to the traditional clustering approaches is Latent Profile Analysis (LPA; Spurk et al., 2020). LPA assumes that the sample can be grouped into a small number of latent profiles and, for a given number of profiles, estimates the mean and variance of each variable within each profile so that the model best fits the observed data. LPA then uses these estimates to calculate, for every person, the probability of belonging to each profile. The profile with the highest probability is usually considered as that person's group, but someone with probabilities of (0.40, 0.35, 0.25) across three profiles can be statistically flagged as having an uncertain or mixed profile (although in practice, many applications still assign such cases to their most probable group).

In the present data, LPA used the seven DASS-21 items directly. Unlike PCA, which first compresses the items into a smaller number of components, LPA estimates profiles in the original item space, so no information is lost through dimensionality reduction. The model in our sample favoured a six-profile solution (Figure S4A). These ranged from a moderately low-symptom group (Profile 4, n = 15) to a severely elevated group (Profile 6, n = 60), with the largest profile representing a mid-severity pattern of elevated fatigue but lower meaninglessness (Profile 3, n = 107). We can also view the probabilistic score of each participant for each cluster (see Figure S4B).

For Review Only

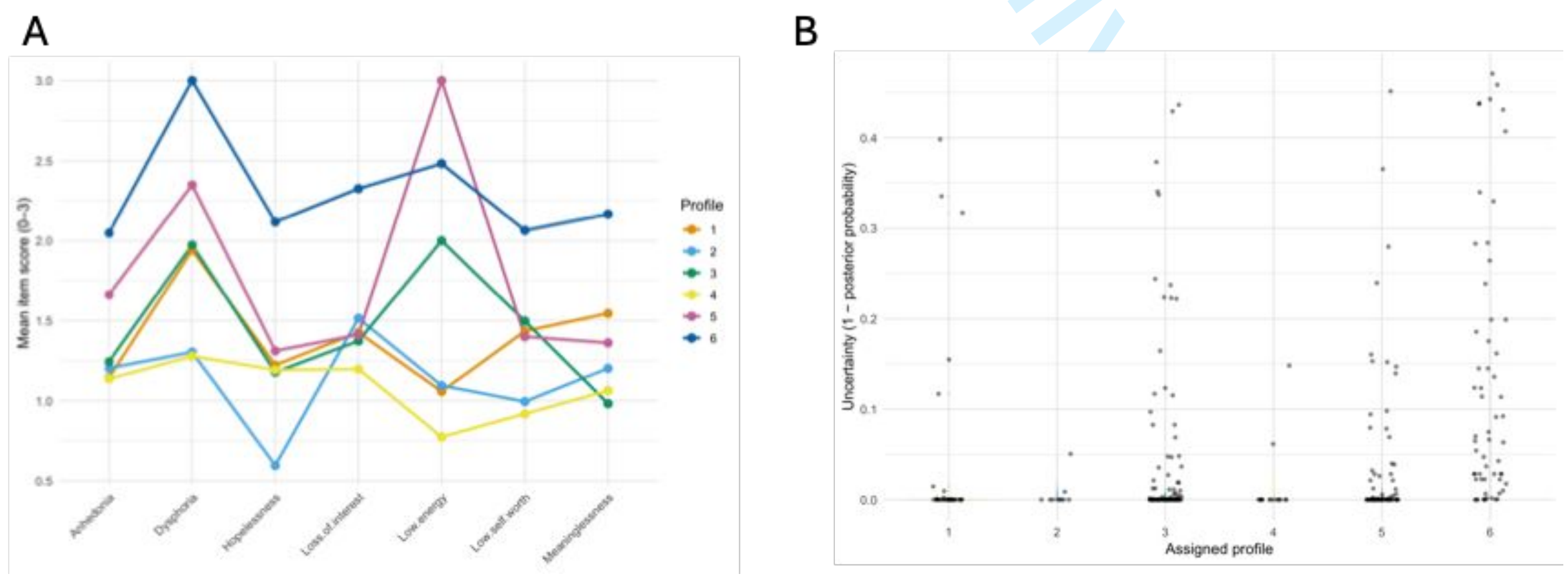

Note. Panel A shows the average symptom profile for each of the six latent profiles. On the x-axis are the seven DASS-21 depression items, and on the y-axis is the mean item score. Each coloured line represents one latent profile. Panel B shows how certain or uncertain the LPA assignments are for

each profile. The x-axis shows the profile to which each person was assigned, and the y-axis shows their uncertainty, calculated as 1 minus their posterior probability for that profile. Points near 0 indicate individuals with high certainty, whose data strongly support membership in their assigned profile. Higher values indicate more ambiguous cases whose responses fit more than one profile reasonably well.

LPA is well-suited when researchers wish to identify distinct subgroups in their data (Spurk et al., 2020). It estimates the defining features of each subgroup and how likely people are to belong to each one. By making ambiguous cases explicit, individuals need not be forced into a single category. In this way, LPA provides a more statistically principled treatment of uncertainty in subgroup membership than k-means clustering (Magidson & Vermunt, 2002). At the same time, this probabilistic assignment remains an indirect means of grouping people. Within the sample, however, each person's profile of observed scores is fixed and not probabilistic, representing the current snapshot of the individual's state (regardless of how accurate or valid the measurement is). To that end, SOMs directly utilise each person's observed profile and arrange people on a continuous map, from which regions of similarity emerge (Kohonen, 2001). Rather than creating a continuum of profiles and then mapping people onto it, SOMs create a continuum of people and then identify patterns within that space.

For Review Only

Additionally, LPA conceptually adopts a discrete, categorical view of the latent structure. Each model is represented by k latent profiles, and each person is assumed to belong (probabilistically) to one of them. When the data are dimensional rather than categorical, as is typically the case for psychiatric symptoms (De Boeck et al., 2023; Haslam et al., 2020), this discrete framing creates a tendency for LPA to compensate by favouring a larger number of profiles to approximate continuous variation. This may help explain the six discrete categories favoured in our case (compared with the three clusters obtained using k-means with PCA and SOMs in the main text). SOMs, in contrast, do not commit to discreteness in advance. The continuous topology of the map

naturally represents both clearly separated regions and graded transitions between them (Kohonen, 2013).

Additionally, the 2D visualisation provided by SOMs allows us to assess the landscape of our construct, and how each profile or region on the map relates to others, which is not something we can see in LPA (Kohonen, 2001). This could be particularly important for example, if we wish to superimpose an additional variable, and see how it maps within current profile (see main text). Lastly, standard LPA assumes within-profile distributions are approximately Gaussian, an assumption rarely satisfied for bounded Likert items with floor and ceiling effects, whereas SOM is non-parametric and makes no such assumption (Kohonen, 2001).

For Review Only

Supplementary References

Beck, A. T. (1996). Manual for the beck depression inventory-II. *(No Title)*.

Borsboom, D. (2017). A network theory of mental disorders. *World Psychiatry*, *16*(1), 5-13. https://doi.org/https://doi.org/10.1002/wps.20375

Borsboom, D., & Cramer, A. O. J. (2013). Network Analysis: An Integrative Approach to the Structure of Psychopathology. *Annual Review of Clinical Psychology*, *9*(Volume 9, 2013), 91-121. https://doi.org/https://doi.org/10.1146/annurev-clinpsy-050212-185608

Bringmann, L. F., Elmer, T., Epskamp, S., Krause, R. W., Schoch, D., Wichers, M., Wigman, J. T. W., & Snippe, E. (2019). *What do centrality measures measure in psychological networks?* [doi:10.1037/abn0000446]. American Psychological Association.

De Boeck, P., Pek, J., Walton, K., Wegener, D. T., Turner, B. M., Andersen, B. L., Beauchaine, T. P., Lecavalier, L., Myung, J. I., & Petty, R. E. (2023). Questioning Psychological Constructs: Current Issues and Proposed Changes. *Psychological Inquiry*, *34*(4), 239-257. https://doi.org/10.1080/1047840X.2023.2274429

Epskamp, S., Borsboom, D., & Fried, E. I. (2018). Estimating psychological networks and their accuracy: A tutorial paper. *Behavior Research Methods*, *50*(1), 195-212. https://doi.org/10.3758/s13428-017-0862-1

Flora, D. B., & Flake, J. K. (2017). The purpose and practice of exploratory and confirmatory factor analysis in psychological research: Decisions for scale development and validation. *Canadian Journal of Behavioural Science / Revue canadienne des sciences du comportement*, *49*(2), 78-88. https://doi.org/10.1037/cbs0000069

Fried, E. I., & Cramer, A. O. J. (2017). Moving Forward: Challenges and Directions for Psychopathological Network Theory and Methodology. *Perspectives on Psychological Science*, *12*(6), 999-1020. https://doi.org/10.1177/1745691617705892

Gao, C. X., Dwyer, D., Zhu, Y., Smith, C. L., Du, L., Filia, K. M., Bayer, J., Menssink, J. M., Wang, T., Bergmeir, C., Wood, S., & Cotton, S. M. (2023). An overview of clustering methods with guidelines for application in mental health research. *Psychiatry Res*, *327*, 115265. https://doi.org/10.1016/j.psychres.2023.115265

Haslam, N., McGrath, M. J., Viechtbauer, W., & Kuppens, P. (2020). Dimensions over categories: a meta-analysis of taxometric research. *Psychological Medicine*, *50*(9), 1418-1432. https://doi.org/10.1017/S003329172000183X

Hoekstra, R. H. A., Epskamp, S., & Borsboom, D. (2023). Heterogeneity in Individual Network Analysis: Reality or Illusion? *Multivariate Behavioral Research*, *58*(4), 762-786. https://doi.org/10.1080/00273171.2022.2128020

Howard, M. C., & Hoffman, M. E. (2018). Variable-Centered, Person-Centered, and Person-Specific Approaches:Where Theory Meets the Method. *Organizational Research Methods*, *21*(4), 846-876. https://doi.org/10.1177/1094428117744021

For Review Only

Jolliffe, I. T., & Cadima, J. (2016). Principal component analysis: a review and recent developments. *Philosophical Transactions of the Royal Society A: Mathematical, Physical and Engineering Sciences*, *374*(2065), 20150202. https://doi.org/10.1098/rsta.2015.0202

Kohonen, T. (2001). *Self-Organizing Maps*. Springer. https://doi.org/10.1007/978-3-642-56927-2

Kohonen, T. (2013). Essentials of the self-organizing map. *Neural Networks*, *37*, 52-65. https://doi.org/10.1016/j.neunet.2012.09.018

Krause, J. S., Reed, K. S., & McArdle, J. J. (2010). Factor Structure and Predictive Validity of Somatic and Nonsomatic Symptoms From the Patient Health Questionnaire-9: A Longitudinal Study After Spinal Cord Injury. *Archives of Physical Medicine and Rehabilitation*, *91*(8), 1218-1224. https://doi.org/https://doi.org/10.1016/j.apmr.2010.04.015

Magidson, J., & Vermunt, J. (2002). Latent class models for clustering: A comparison with K-means. Canadian J Mark Res. *Professional Marketing Research Society*, *20*, 36-43.

Mazziotta, M., & Pareto, A. (2019). Use and Misuse of PCA for Measuring Well-Being. *Social Indicators Research*, *142*(2), 451-476. https://doi.org/10.1007/s11205-018-1933-0

R Core Team. (2021). *R: A language and environment for statistical computing*. https://www.R-project.org/

Revelle, W. (2026). Package 'psych'.

Scrucca, L., Fop, M., Murphy, T. B., & Raftery, A. E. (2016). mclust 5: Clustering, Classification and Density Estimation Using Gaussian Finite Mixture Models. *R j*, *8*(1), 289-317.

Spurk, D., Hirschi, A., Wang, M., Valero, D., & Kauffeld, S. (2020). Latent profile analysis: A review and "how to" guide of its application within vocational behavior research. *Journal of Vocational Behavior*, *120*, 103445. https://doi.org/https://doi.org/10.1016/j.jvb.2020.103445

For Review Only

Watkins, M. W. (2018). Exploratory Factor Analysis: A Guide to Best Practice. *Journal of Black Psychology*, *44*(3), 219-246. https://doi.org/10.1177/0095798418771807

For Review Only
